# Myricetin Possesses Anthelmintic Activity and Attenuates Hepatic Fibrosis *via* Modulating TGFβ1 and Akt Signaling and Shifting Th1/Th2 Balance in *Schistosoma japonicum*-Infected Mice

**DOI:** 10.3389/fimmu.2020.00593

**Published:** 2020-04-16

**Authors:** Ping Huang, Minyu Zhou, Shaoyun Cheng, Yue Hu, Minzhao Gao, Yubin Ma, Yanin Limpanont, Hongli Zhou, Paron Dekumyoy, Yixin Cheng, Zhiyue Lv

**Affiliations:** ^1^Joint Program of Pathobiology, Fifth Affiliated Hospital, Zhongshan School of Medicine, Sun Yat-sen University, Guangzhou, China; ^2^Ministry of Education, Key Laboratory of Tropical Disease Control, Sun Yat-sen University, Guangzhou, China; ^3^Key Laboratory of Tropical Translational Medicine of Ministry of Education, Hainan Medical University, Haikou, China; ^4^Guangdong Provincial Key Laboratory of Biomedical Imaging, Fifth Affiliated Hospital, Sun Yat-sen University, Zhuhai, China; ^5^Faculty of Tropical Medicine, Mahidol University, Bangkok, Thailand

**Keywords:** *Schistosoma japonicum*, myricetin, Th1/Th2 balance, TGFβ1/Smad, Akt

## Abstract

Schistosomiasis is a zoonotic and debilitating parasitic disease caused by *Schistosoma japonicum*. Praziquantel remains the choice for treating schistosomiasis, but its efficacy could be hampered by emergence of resistance. In this study, using large-scale drug screening, we selected out myricetin, a natural flavonol compound, having a good anti-schistosome effect. We found that myricetin exhibited dose and time-dependent insecticidal effect on *S. japonicum in vitro*, with an LC_50_ of 600 μM for 24 h, and inhibited female spawning. The drug mainly destroyed the body structure of the worms and induced apoptosis of the worm cells, which in turn led to death. In addition, oral administration of myricetin in mice infected with *S. japonicum* showed a deworming effect *in vivo*, as evidenced by a significant reduction in the liver egg load. H&E staining, quantitative RT-PCR, and Western blotting assays showed that myricetin significantly alleviated liver fibrosis in mice infected with *S. japonicum*. Myricetin also effectively inhibited the expression of TGFβ1, Smad2, phospho-Smad2, Smad3, phospho-Smad3, ERK, phospho-ERK, Akt, and phospho-Akt in the liver of infected mice, suggesting that myricetin attenuated liver fibrosis in mice *via* modulating TGFβ1 and Akt signaling. Flow cytometric analysis of Th subtypes (Th1/Th2/Th17/Treg) in the mouse spleen further revealed that myricetin significantly increased the percentage Th1 cells in infected mice and reduced the proportion of Th2 cells and Th17 cells. Immunology multiplex assay further showed that myricetin attenuated *S. japonicum*-induced rise in the plasma levels of IL-4, IL-5, IL-10, IL-13, and IL-17A in infected mice while increasing the plasma contents of IFN-γ, IL-12, and IL-7. In conclusion, our study provides the first direct evidence that myricin possesses potent anti-schistosome activities *in vitro* and *in vivo*, and offers new insights into the mechanisms of action by myricetin. The present findings suggest that myricetin could be further explored as a therapeutic agent for *S. japonicum*.

## Introduction

Schistosomiasis is a parasitic disease caused by parasites of the genus *Schistosom*a. According to a World Health Organization survey, schistosomiasis, one of the neglected tropical diseases, is endemic in 78 countries and regions, infecting ~230 million people and posing a health threat to ~780 million people worldwide. Among them, 120 million infections are symptomatic, and nearly 300,000 deaths occur due to schistosomiasis annually in the sub-Saharan Africa area (1–3). There are mainly six species of schistosomiasis that are related to humans, of which *Schistosoma* (*S*.) *mansoni, S. haematobium*, and *S.japonicum* are the most prevalent (4). The life expectancy of adult schistosomes is on the average 3–10 years in the human host, and in some cases, it can be as long as 40 years (5, 6). Although schistosomiasis cannot proliferate in the final host, it can produce a large number of eggs deposited in the liver or other organs. Mature females can lay hundreds (*S. mansoni, S. haematobium*) to thousands (*S. japonicum*) of eggs per day. Long-term parasitism and massive egg production leads to infection and disease transmission.

In the absence of effective vaccines, chemotherapy is an important measure for schistosomiasis control. Currently, the drug of choice for clinical use is praziquantel, which is effective against all schistosome species in humans and has low toxicity (7–9). However, the application of praziquantel has some limitations. For example, it can kill early schistosomula (3–6 h) that have just penetrated into the skin and adult worms, but it has a weak effect on schistosomula and no preventive effect (10). Repeated infections after treatment is another major problem in schistosomiasis control. Meanwhile, due to the long-term and large-scale repeated uses of praziquantel in endemic areas, resistance to praziquantel may be emerging (11, 12). British scholars have induced a praziquantel-resistant *S. manoni* strain using sub-dose praziquantel to treat infected mice (13), so it is urgent to develop an alternative drug.

Currently, there are three ways to develop praziquantel alternatives (14): synthesis of new praziquantel derivatives, design of new pharmacophores and large-scale screening of new compounds. In this study, we obtained myricetin (3, 3′, 4′, 5, 5′, 7-hexahydroxy flavone), a compound with potential effects on *S. japonicum*, through large-scale screening of small-molecule compound libraries. Myricetin is a natural flavonol compound and widely exists in many natural plants, fruits, and vegetables (15), and has a wide range of pharmacological activities, including anti-oxidant, anti-tumor, anti-inflammatory, anti-microbial, anti-allergic, cardiovascular, and neuronal protection effects (15). Recent studies have revealed that myricetin can improve CCl4-induced liver fibrosis in mice (16), but its effect on liver disease caused by *S. japonicum* infection is yet to be determined.

In this study, we observed the anti-*S. japonicum* adult effect of myricetin *in vitro*, and then a mouse model of *S. japonicum* cercariae infection was established and treated with myricetin. Pathological damage and expression of liver fibrosis factors in infected mice before and after treatment were detected and its underlying mechanism was explored in order to evaluate the potential value of myricetin as a novel anti-*S. japonicum* drug.

## Materials and Methods

### Ethics Statement

The Institutional Animal Care and Use Committee of Sun Yat-sen University approved all animal experiments in this study (No. 2019-2663 and No. 2019-070). Animals were maintained under specific pathogen-free conditions with unrestricted access to sterilized food and water.

### Animals

*S. japonicum*-infected *Oncomelania* (*O*.) *hupensis* were supplied by the National Institute of Parasitic Diseases, Chinese Center for Disease Control and Prevention, Shanghai, China. New Zealand rabbits (2.0–2.5 kg) and BALB/c mice (6–8 weeks) (Charles River, Beijing, China) were maintained in a specific pathogen-free environment and had *ad libitum access* to water and food.

The study protocol for all animal experiments was approved by The Institutional Animal Care and Use Committee of Sun Yat-sen University. Animal studies were carried out in strict accordance with institutional and state guidelines on the use of experimental animals.

### Drugs

The small molecule compound library was donated by Dr. Kai Deng at Sun Yat-sen University. Myricetin and dimethyl sulfoxide (DMSO) were purchased from Sigma-Aldrich (St. Louis, MO, USA), and RPMI 1,640 medium, penicillin/streptomycin and fetal bovine serum were purchased from Gibco (California, USA). Praziquantel tablets (Nanjing Pharmaceutical Factory Co., Ltd., Nanjing, China) were gifts from Dr. Shouyi Chen at Guangzhou Center for Disease Control and Prevention, China.

### Animal Infection

At an ambient temperature of 25 ± 1°C, *O. hupensis* were put into a 12-well plate, and after addition of dechlorinated water to a 2/3 volume, they were placed under an incandescent lamp for 2 h for cercaria escape. Then, the abdominal fur of rabbits and mice was shaved and the skin moistened with dechlorinated water. The cercariae were counted on a cover slip and then attached to the depilated skin of the animals. After 20 min, the slide was removed. Each New Zealand rabbit was infected with 1000–1200 cercariae, and each mouse was infected with 30 ± 2 cercariae.

### *In vitro* Insecticidal Experiments

#### Drug Screening

At 8 weeks post-infection, New Zealand rabbits were sacrificed by air embolization after blood was taken from the heart, and adult *S. japonicum* worms parasitizing in the mesenteric vein and hepatic portal vein were collected after dissection. After wash with normal saline, the worms were put into a 24-well plate, and each well-contained 3 pairs of adults/1 mL complete medium (RPMI 1,640 medium containing 100 U/ml penicillin, 100 μg/ml streptomycin and 10% heat-inactivated serum), and then placed in the incubator (37°C, 5% CO_2_) for 4 h. Then, different small-molecule drugs (1,000 μM) were added, with 100 μM praziquantel and 1% DMSO as positive and negative control, respectively. At 24, 48, and 72 h of incubation, the survival status of the parasites was evaluated under an inverted microscope and its viability was scored (17) to screen out the drug with obvious insecticidal effect.

#### Activity of Myricetin Against *S. japonicum in vitro*

Like the drug screening method, in each well with 6 males or females, 1 mL complete medium containing myricetin at different concentrations (300, 400, 500, 600, 700, and 800 μM) was added, with 100 μM praziquantel and 1% DMSO as positive and negative control, respectively. At different time points (24, 48, 72, and 96 h), the survival status of the worms was observed under the microscope and their viability was scored. The culture medium of each group was collected after 96 h incubation, and then centrifuged. The supernatant was discarded, and 1 ml pre-chilled PBS (pH 7.4) was added to resuspend the precipitates. The number of eggs in each group was counted. Three independent experiments were performed; unless otherwise stated, after incubation with the half-lethal dose (LC_50_) of myricetin, 100 μM praziquantel (positive group) and 1% DMSO (negative group) for 24 h, the worms were collected for subsequent experiments after wash with normal saline, such as acetocarmine-fast green staining, scanning electron microscopy (SEM), transmission electron microscopy (TEM), real-time quantitative polymerase chain reaction (RT-qPCR) and flow cytometric analysis.

#### Acetocarmine-Fast Green Staining

Adult worms were fixed with 95% ethanol, 3% formalin and 2% glacial acetic acid solution overnight, followed by transfer to acetic acid magenta solution for 1–2 h at room temperature, and separation with 1% HCl. The worms were dehydrated with 80 and 90% ethanol for 5 min, respectively, and then transferred to a solid green staining solution for several seconds. Finally, the worms were transparentized with methyl salicylate for 24 h, and was sealed with neutral gum, and observed under an inverted microscope and photographs were taken (Leica, Heidelberg, Germany).

#### Scanning Electron Microscope

Adult worms were fixed with 2.5% glutaraldehyde for 24 h. The fixed specimens were dehydrated in gradient ethanol (50, 70, 85, and 100%) for 5–10 min, respectively, and replaced with pure acetone for 15–20 min, and then with isoamyl acetate for 15–30 min. The worms were transferred carefully to the sample cage, dried at critical point, coated with gold on surface, and observed by HITACH2S570 (HITACHI, Tokyo, Japan).

#### Transmission Electron Microscope

After washing of the worms three times with PBS, the middle part of worms was taken using a surgical blade under a stereo microscope (SZ650, Cnoptec, Chongqing, China), and put in 2.5% glutaraldehyde overnight at 4°C, and then fixed with 1% osmic acid for 1 h. After gradient acetone dehydration, the specimen was dehydrated twice with pure acetone and embedded in 812 epoxy resin (Ted Pella Inc., Redding, USA). After ultra-thin sectioning (60 nm), the slices were immersed in uranyl acetate-lead citrate for double staining and viewed under a Tecnai G2 Spirit Twin electron microscope (FEI, Hillsboro, USA) operated at 80 kV.

In addition, after anesthesia by intraperitoneal sodium pentobarbital, mouse heart in each group was perfused with normal saline, and then fixed with a mixture of 2.5% glutaraldehyde and 4% paraformaldehyde, and then the left lobe of the liver was morselized (1 mm × 1 mm × 1 mm) and immersed in the fixative solution, and then viewed with a transmission electron microscope.

#### Real-Time Quantitative RT-PCR

Total RNA of *S. japonicum* was extracted using TRIzol Reagent (Thermo Fisher Scientific, Waltham, USA) as instructed by the manufacturer. Then, cDNA was synthesized from total RNA using the RevertAid First Strand cDNA Synthesis kit (Thermo Fisher Scientific). The primers of 13 apoptosis-related genes are displayed in Table 1. *NADH* was used as the internal reference gene. In addition, total RNA of liver tissues was extracted. Liver fibrosis-related gene primers are shown in Table 2. *GAPDH* served as the internal reference gene. Real time quantitative PCR (RT-qPCR)was performed using SYBR® Premix Ex Taq ™ (Takara, Tokyo, Japan) in a 20- μL volume. The PCR was run on a real-time quantitative PCR system (Bio-Rad, California, USA) at 95°C for 30 s, followed by 95°C for 5 s and 60°C for 34 s for 40 cycles. Next, the melting curve was analyzed (95°C for 15 s and 65°C for 15 s.). The relative expression of each gene was calculated using the 2^−ΔΔCt^ method (18).

**Table 1:** The primer sequences of *Schistosoma japonicum* apoptosis-associated genes used for quantitative real-time PCR.

**Gene**	**Forward primer sequence**	**Reverse primer sequence**
Bak	GCATCAGCCAAGGCATCTTCACAA	TCAACACTATGTGGTTCAGCCCGA
Bax	GGAGACAATGGAGACCGAAA	CGACCAATTAGGGCTTGTGT
Bcl-2	TGACTGTTACTACTCGCTCTG	TGACTCACAATCTCGCATG
CYC	CCACACAAAACAGGACCCAA	CCTTCTTCAAACCAGCAAATACC
Caspase 2	TGCTAGCTGGGAAACCCAAG	TTCACGAGAATTCGACGGCA
Caspase 3	ATCGCTGTCCTTCCCTGATTGGAA	ATTACATCATCGCCTGCATCGGCA
Caspase 7	TGACGTGCAAAATATTAAGAGAGCC	CCACCTTCATCACCATGAGAAA
Caspase 9	GGGTGAAGAACGCAATCATAAC	CCAAGAAACAAATCCAGGCAAA
APAF	TCTGGATCCCACCGTTTACCAACT	AGCACTCGTCCAACTTCAACATCC
CIAP	GCGGCCGTTTCCACTCATGTTAAA	ACCACCGGGTTGTAAACAGGATGA
IAP	TCCGCCTACAAAGTCAATCTAC	TCACTACCTTCGCTCAATGC
AIF	TGCCGAATTAGCCTACTGGTGTCT	TGGTGGTGGTGTAGAATCCTTGT
API	TCTGTGCTGTCAACTTGG	GCGTAATCTTGTGCTAACTG
NADH	CGAGGACCTAACAGCAGAGG	TCCGAACGAACTTTGAATCC

**Table 2:** The primer sequences of liver fibrosis related genes (mice) used for quantitative real-time PCR.

**Gene**	**Forward primer sequence**	**Reverse primer sequence**
α-SMA	CACAGCCCTGGTGTGCGACAAT	TTGCTCTGGGCTTCATCCCCCA
Collagen I	GGAGACAATGGAGACCGAAA	CGACCAATTAGGGCTTGTGT
Collagen IV	ATGCCCTTTCTCTTCTGCAA	GAAGGAATAGCCGATCCACA
TGF-β1	CAACAATTCCTGGCGTTACCTTGG	GAAAGCCCTGTATTCCGTCTCCTT
TGF-β1RII	TACGAGCCCCCATTTGGTTC	CCAGCACTCGGTCAAAGTCT
Smad2	GTATGGACACAGGCTCTCCG	ACCAGAATGCAGGTTCCGAG
Smad3	CTCCAAACCTCTCCCCGAAT	GAGTTGGAGGGGTCAGTGAA
ERK	ACCACATTCTAGGTATCTTGGGT	AGTTTCGGGCCTTCATGTTAAT
GAPDH	CAGATCCACAACGGATATATTGGG	CATGACAACTTTGGCATTGTGG

#### Flow Cytometry

For examination of apoptosis of *S. japonicum*, male and female *S. japonicum* were collected separately, and digested with 4% trypsin-EDTA for 4 h at 4°C, and the mixture was gently suspended every 30 min, and then filtered through a 70 μm cell sieve. After centrifugation at 600 g for 10 min, the supernatant was discarded and the pellet was resuspended into a single cell suspension with pre-chilled PBS (pH 7.4). *S. japonicum* was stained using FITC-Annexin V/propidium iodide (BD Biosciences, Franklin Lakes, USA) and apoptosis was examined using a CytoFLEX flow cytometer (Beckman Coulter, Atlanta, USA).

For examination of cytokine production, mouse spleen was harvested and rendered into single cell suspensions. Red blood cells were lysed and the cell suspension was adjusted to an appropriate concentration. Lymphocytes were stimulated with phorbol myristate acetate/ionomycin (Multisciences, Hangzhou, China) for 4 h, and then Golgi inhibitor brefeldin A/monensin (Multisciences) was added to prevent the exocytosis of cytokines. FITC-conjugated anti-CD3e and V450-conjugated anti-CD8 antibody (BD Biosciences) were added for surface staining, and then the cells were resuspended with fixation/permeabilization buffer (BD Biosciences) and PE-conjugated anti-IFN-γ, IL-17A, APC-conjugated anti-IL-4 antibodies (BD Biosciences) were added for intracellular cytokine staining.

For detection of Treg cells, V450-conjugated anti-CD4 and BB515-conjugated anti-CD25 antibodies (BD Biosciences) were added for surface staining, and then fixed and permeabilized with foxp3 staining buffer (Invitrogen) according to the instructions. Cells were incubated for 30 min at 4°C in the dark for 40–50 min with AF647-conjugated anti-Foxp3 antibody, and then detected with a CytoFLEX S flow cytometer.

#### Anti-Schistosome Effect *in vivo*

##### Schistosome-infected mice treated with myricetin in vivo

BALB/c mice were randomly divided into four groups: the control group, the infected group, the praziquantel-treated group and the myricetin-treated group (8 mice in each group). Except for the control group, the remaining mice were infected with 30 ± 2 *S. japonicum* cercariae as described above. Thirty-six to 42 days after infection, the mice in the praziquantel-treated and me-treated groups were administered with praziquantel 500 mg/kg and myricetin 250 mg/kg (dissolved in 0.9% normal saline) daily, and the other two groups received the same volume of saline by gastric lavage. At week 7 post *S. japonicum* infection (wpi), the mice in each group were weighed, and then anesthetized with pentobarbital sodium intraperitoneally, and blood samples were drawn from the orbit. The adult worms were collected from the hepatic portal vein and mesenteric vein *via* cardiac perfusion, and liver and spleen tissues were collected, weighed and then kept according to different experimental needs. All the worms were placed in a 60 mm Petri dish containing normal saline, and the total number of worms, the number of females and males were counted under a stereo microscope (19, 20).

##### Egg count

The median liver lobe in mice of each group was partially cut out, weighed and placed in an Eppendorf tube, and morsellized, and then added with 4% potassium hydroxide solution, and shaken in a 37°C constant temperature shaker overnight till the liver tissue was completely digested. After centrifugation at 1,500 g for 5 min, the supernatant was removed, and the precipitates were dissolved in 1 mL normal saline and 10 μl of the resuspension with eggs was counted manually on a microscopic slide. The experiment was repeated 5–7 times. The number of worm eggs per gram of liver tissue was calculated (21).

##### Enzyme-Linked Immunosorbent Assay (ELISA)

Whole blood was collected from the mice and kept at room temperature for 30 min, and after coagulation, it was centrifuged at 4,000 rpm for 20 min. The serum was harvested, and type III procollagen (PC III), collagen IV, laminin and hyaluronidase were measured by ELISA Kit (Cusabio, Wuhan, China).

##### Histopathological examination

The left liver tissue of the mice was partially collected and immersed in 4% paraformaldehyde for 24 h, and then the tissue was embedded in paraffin and sectioned. Tissue sections were dewaxed in xylene, and then dehydrated in gradient alcohol, and stained according to haematoxylin-eosin (H&E) and Masson staining instructions (Biosharp, Wuhan, China). Pathological changes were observed under a full automatic upright microscope (Olympus, Tokyo, Japan). The area of granulomas was calculated as previously described (22), and the area of liver fibrosis was measured using Image Pro plus 6.0 software (MEDIA CYBERNETICS image technology Inc., Maryland, USA).

##### Western blotting assay

Liver tissues were lyzed with RIPA lysis buffer (Thermo Fisher Scientific) or NP40 lysis buffer (Beyotime, Shanghai, China). The lysates were resolved by 12% SDS-polyacrylamide gel electrophoresis. The detection of Collagen I and Collagen IV molecules needs to be performed under non-denaturing conditions. Primary antibodies against the following proteins were used: TGFβ1, SMA, collagen I and IV, Smad2 and phospho-Smad2, Smad3 and phospho-Smad3, ERK1/2 and phospho-ERK1/2, and GAPDH (Abcam, Cambridge, UK). After incubation with a HRP-conjugated goat anti-rabbit IgG (Abcam), the protein bands were detected using Pierce Enhanced Chemiluminescence Detection Reagent (Millipore, Burlington, USA) on a chemiluminescence imaging system (Bio-Rad) and quantified using Image J (National Institutes of Health, Bethesda, USA).

##### Immunology multiplex assay

Levels of cytokines in mouse serum were determined using Mouse High Sensitivity T Cell Magnetic Bead Panel as instructed by the manufacturer (Millipore, Massachusetts, USA).

##### Data analysis

Quantitative data was expressed as means ± standard deviation (SD). Differences between groups were analyzed by one-way analysis of variance (ANOVA) using SPSS 19.0 (SPSS Inc, New York, USA). All histograms were drawn by GraphPad Prism 7.0 (GraphPad Software, San Diego, USA). *P* < 0.05 was considered statistically significant.

## Results

### Myricetin Exhibits Dose- and Time-Dependent Insecticidal Effect on *S. japonicum in vitro* and Inhibits Female Spawning

**Figure 1:**
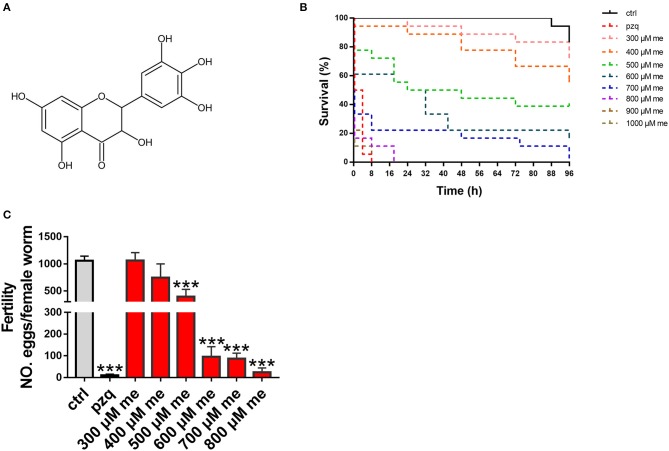
*In vitro* effects of myricetin on the activity of *S. japonicum*. **(A)** Chemical structure of myricetin (C_15_H_10_O_8_, MW318.24) **(B)** Insecticidal activity myricetin against *S. japonicum* in different time intervals and concentrations in the laboratory **(C)** Pairs of adult worms were treated with myricetin in different concentrations (300, 400, 500, 600, 700, and 800 μM) for 96 h, and the number of eggs laid in culture was counted under an inverted microscope. As positive pzq and negative control ctrl group, adult worms were incubated in RPMI 1,640 medium with praziquantel (100 μM) or 1% DMSO, respectively. Data were shown as the means ± SD (*n* = 3). ^***^P < 0.001, compared with the negative ctrl group.

Totally 480 small-molecule compounds in the compound library (Supplementary Table 1) were screened to determine their effects on the viability of *S. japonicum*. The initial detection concentration was 1,000 μM. In this compound library, myricetin had obvious helminthicidal effect *in vitro* (Figure 1A). To further delineate the effect of myricetin on schistosomes, we examined the effect of myricetin at different concentrations on the worms over time under a microscope. Myricetin exhibited a time- and dose-dependent helminthicidal effect on adult *S. japonicum in vitro*, with an LC_50_ of 600 μM for 24 h. All the worms died by 8 h post-treatment with myricetin (1,000 μM) or praziquantel while about 80% of the untreated worms were still alive at 96 h (Figure 1B, Table 3).

**Table 3:** The time- and dose- dependent effects of myricetin on *S. japonicum* adult worms.

**C (μmol/L)**	**Number of worms**	**24 h**	**48 h**	**72 h**	**96 h**
		**score/VDR (%)**	**score/VDR (%)**	**score/VDR (%)**	**score/VDR (%)**
**Male**					
Ctrl	6	18.0 ± 0.0/0.0	18.0 ± 0.0/0.0	18.0 ± 0.0/0.0	16.7 ± 1.2/7.0
Pzq	6	0.0 ± 0.0/100.0	0.0 ± 0.0/100.0	0.0 ± 0.0/100.0	0.0 ± 0.0/100.0
me (300)	6	16.7 ± 1.2/7.0	16.7 ± 1.2/7.0	16.7 ± 1.2/7.0	14.0 ± 2.0/22.0
me (400)	6	12.7 ± 1.2/30.0	12.7 ± 1.2/30.0	10.0 ± 2.0/44.0	8.0 ± 3.5/56.0
me (500)	6	8.7 ± 1.2/52.0	7.3 ± 1.2/59.0	3.3 ± 1.2/81.0	0.3 ± 0.6/98.0
me (600)	6	7.3 ± 1.2/59.0	3.3 ± 2.3/81.0	0.7 ± 1.2/96.0	0.0 ± 0.0/100.0
me (700)	6	5.3 ± 1.2/70.0	4.7 ± 1.2/74.0	1.0 ± 1.0/94.0	0.0 ± 0.0/100.0
me (800)	6	0.0 ± 0.0/100.0	0.0 ± 0.0/100.0	0.0 ± 0.0/100.0	0.0 ± 0.0/100.0
**Female**					
Ctrl	6	18.0 ± 0.0/0.0	18.0 ± 0.0/0.0	18.0 ± 0.0/0.0	16.3 ± 1.5/9.0
Pzq	6	0.0 ± 0.0/100.0	0.0 ± 0.0/100.0	0.0 ± 0.0/100.0	0.0 ± 0.0/100.0
me (300)	6	18.0 ± 0.0/0.0	16.7 ± 2.3/7.0	16.0 ± 2.0/11.0	14.7.0 ± 1.2/19.0
me (400)	6	14.7 ± 1.2/19.0	12.7 ± 2.3/30.0	11.3 ± 1.2/37.0	10.7 ± 3.1/41.0
me (500)	6	12.0 ± 0.0/33.0	10.7 ± 1.2/41.0	9.3 ± 3.1/48.0	2.7 ± 1.2/85.0
me (600)	6	10.0 ± 0.0/44.0	9.3 ± 5.0/48.0	4.7 ± 1.2/74.0	0.7 ± 1.2/96.0
me (700)	6	9.3 ± 1.2/48.0	4.0 ± 2.0/78.0	2.7 ± 1.2/85.0	0.0 ± 0.0/100.0
me (800)	6	0.0 ± 0.0/100.0	0.0 ± 0.0/100.0	0.0 ± 0.0/100.0	0.0 ± 0.0/100.0

*Adult worms were incubated with myricetin in different concentrations. The viability of worms were scored at 24, 48, 72, and 96 h under inverted microscope using a viability scale of 0–3 (3, normally active; 2, slow activity; 1, minimal activity, occasional movement of head or tail; 0, total absence of mobility). ctrl, adult worms were incubated in RPMI 1,640 medium with 1% DMSO. pzq, praziquantel (100 μM) was used as positive control groups. VDR, Vitality Decrease Rate*.

*Schistosoma* eggs are recognized as the leading cause of liver damage in the host (23). When adult worms were incubated with myricetin at different concentrations for 96 h (end point), the number of eggs in each group was counted. When the concentration of myricetin was <500 μM, egg production by females did not significantly differ from that of the normal control group (*P* > 0.05). Meanwhile, egg production decreased to 394.3 ± 136.6 with 500 μM myricetin [*F*_(9, 20)_ = 50.26, *P* < 0.001] and 24.67 ± 19.4 with 800 μM myricetin [*F*_(9, 20)_ = 50.26, *P* < 0.001], showing a dose-dependent decrease in the female spawning rate (Figure 1C).

### Myricetin Damages the Integrity of the Tegument of *S. japonicum*

**Figure 2:**
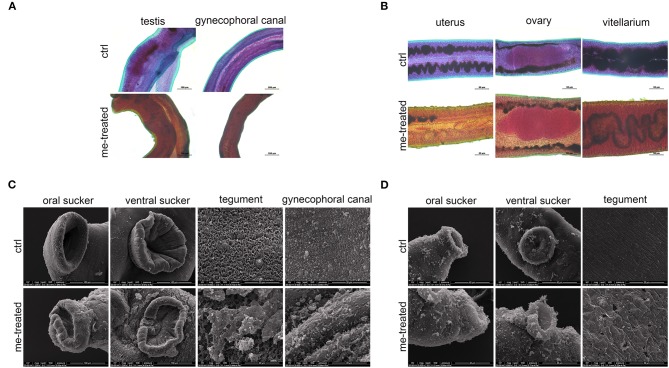
Morphological changes in male and female of *S. japonicum in vitro*. Adult worm pairs were incubated with myricetin (me) at 600 μM for 24 h, and female and male worms were separated for analysis. Adult male **(A)** and female **(B)** were stained with acetocarmine-fast green and observed under Leica microscope. **(C)** Scanning electron microscopy (SEM) analysis of the oral sucker, ventral sucker, tegument and the special structure of male worms, gynecophoral canal of *S. japonicum* following treatment with myricetin. Scale bars: **(A)** upper left row, 100 μm; lower right row, 200 μm **(B)** 50 μm and **(C)** The oral sucker and ventral sucker of male worms, 100 μm; **(D)** The oral sucker and ventral sucker of female worms, 40 μm; The tegument and gynecophoral canal, 20 μm. ctrl, adult worms were incubated in RPMI 1,640 medium with 1% DMSO; me-treated, adult worms treated with myricetin.

The male worm body became stiff with wrinkles on the tegument, and the intestinal branches became disordered. Vesicle-like protrusions appeared on tegument of the female worm, and the intestine ruptured leaking its contents. Some worms' ovaries were irregular in shape and stained unevenly (Figures 2A,B). SEM further revealed that upon treatment with myricetin, the male worms showed a depressed oral and ventral sucker with a swollen margin, and its surface displayed irregular contraction and protuberances deformation. Ridges were arranged irregularly, with loss of cilia and collapse of sensory papillae. Necrosis and rupture appeared at the edge of the male worms' oral sucker and ventral sucker adhering to a lump. The ridges fused together to form a mass in the middle and caudal portion of the integument with decreased sensory papillae. These results indicated that myricetin can damage the integrity of the tegument of *S. japonicum* (Figures 2C,D).

### Myricetin Promotes Apoptosis of the Worms

**Figure 3:**
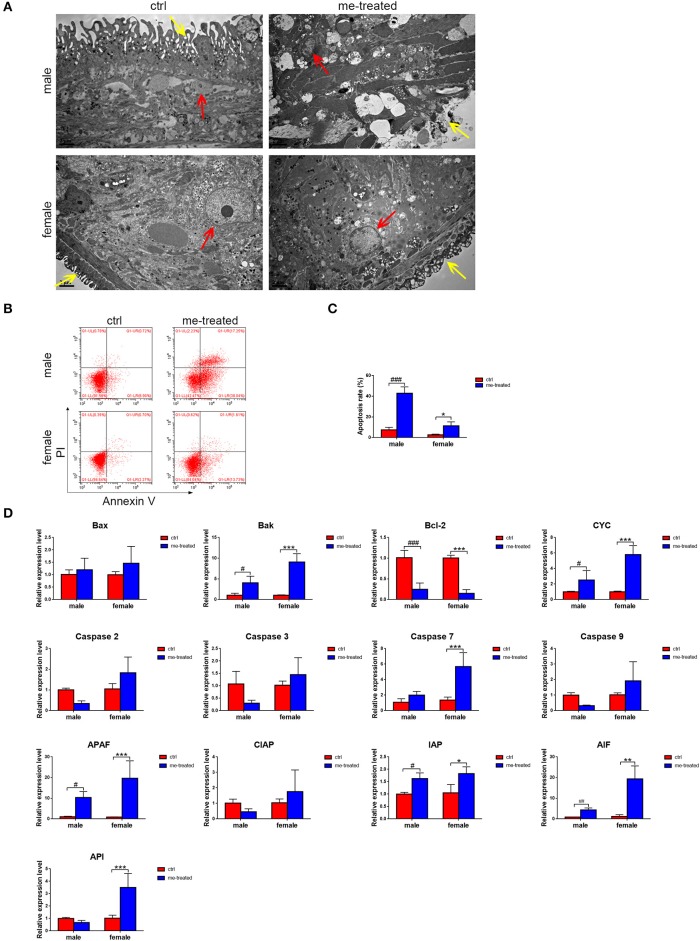
Myricetin induces the apoptosis process and destroy the tegument architecture of *S. japonicum* adult worms *in vitro*. **(A)** Adult worms following treatment with myricetin were observed by transmission electron micrographs (TEM). The yellow and red arrows indicate tegument and cell nucleus, respectively. (Scale bars: 2 μm) **(B)** Apoptosis induced with myricetin was detected using Flow cytometry in worms. **(C)** Apoptosis induced with myricetin was statistical analyzed in worms. **(D)** Relative expression levels of 13 apoptosis associated genes after treatment with myricetin measured by Real-time qPCR. Data were shown as the means ± SD. ^#^*P* < 0.05, ^##^*P* < 0.01, ^###^*P* < 0.001, compared with the negative ctrl group of male worms; ^*^P < 0.05, ^**^P < 0.01, ^***^P < 0.001, compared with the negative ctrl group of female worms. Data are representative results from at least 15 worms investigated in three independent experiments. ctrl, adult worms were incubated in RPMI 1,640 medium with 1% DMSO; me-treated, adult worms treated with myricetin.

TEM revealed tegument peeling, disorganized tegument structure and vesicles between the tegument matrix and parenchymal cells in male worms in the myricetin-treated group, and a rough outer plasma membrane and swollen myofilaments in female worms. Worm cells treated with myricetin exhibited apoptosis related morphological changes, mainly including karyorrhexis, pyknosis, cytoplasm concentration and increased compactness and cytoplasmic vacuolation (Figure 3A). Flow cytometry further revealed that treatment with myricetin for 24 h caused a 5.8- and 4.0- fold increase in the percentage of apoptotic cells of the male (myricetin: 42.7 ± 6.5% *vs*. control: 7.4 ± 2.6%; *t* = 8.776, *P* = 0.0009) and the female worms (myricetin: 11.3 ± 2.3% vs. control: 2.82 ± 0.3; *t* = 3.730, *P* = 0.0203), respectively. These findings suggest that both male and female worms are sensitive to myricetin (Figures 3B,C).

Our quantitative RT-PCR assays demonstrated that compared with the control group, the expression of *Bak*, cytochrome c (*CYC*) and apoptotic protease activating factor 1 (*APAF*) in males increased 4-fold (*t* = 2.836, *P* = 0.022), 2.8-fold (*t* = 3.231, (*P* = 0.012) and 10-fold (*t* = 2.521, *P* = 0.036), respectively, while the expression of *Bak, CYC, caspase 7, APAF, AIF*, and *API* in females increased 9-fold (*t* = 7.566, *P* < 0.001), 5.8-fold (*t* = 8.509, *P* < 0.001), 4.2-fold (*t* = 5.363, *P* = 0.001), 22.8-fold (*t* = 5.101, *P* = 0.001), 15.8-fold (*t* = 6.952, *P* < 0.001) and 3.5-fold (*t* = 5.15, *P* = 0.001), respectively. The expression of anti-apoptotic gene *Bcl-2* decreased 4-fold (*t* = 7.122, *P* < 0.0001) and 6.7-fold (*t* = 7.982, *P* < 0.001) in males and females, respectively (Figure 3D). The results confirmed that myricetin induces apoptosis of *S. japonicum*.

### Myricetin Reduces Egg Production of *S. japonicum* and Adult Worm Load in Mice

Mice were treated with 500 mg/kg praziquantel or 250 mg/kg myricetin daily after infection with *S. japonicum* by intragastric lavage for 35–42 days. The mice were euthanized at 7 weeks after infection. The results showed that myricetin significantly reduced the adult worm load in mice [infected: 14.4 ± 2.6; myricetin: 5.6 ± 2.1; *F*_(3, 16)_ = 83.32, *P* < 0.001]. Interestingly, the egg load in the mouse liver in each group indicated that myricetin effectively inhibited egg production of *S. japonicum* [eggs per gram of liver tissue: infected: 19626.8 ± 3226.1 *vs*. myricetin: 5727.6 ± 1786.0; *F*_(3, 16)_ = 102.2, *P* < 0.001], and their inhibitory effects were not significantly different (*P* > 0.05) (Table 4).

**Table 4:** Worm and egg burden in liver after treatment of *S. japonicum* infected BALB/c mice with praziquantel or myricetin.

**Group**	**Total worms**	**Male worms**	**Female worms**	**No. of eggs found in liver, per gram**
Ctrl	neg	neg	neg	neg
Infected	14.4 ± 2.61	7.6 ± 1.14	6.8 ± 1.64	19626.81 ± 3226.05
pzq-treated	neg	neg	neg	1238.09 ± 1495.85^***^
me-treated	5.6 ± 2.07^***^	3.8 ± 1.79^**^	1.8 ± 0.45^***^	5727.63 ± 1786.04^***^

*Balb/c mice were infected percutaneously with 30 ± 2 cercariae of S. japonicum. Worm and egg burdens were determined at 7 weeks post-infection. Data are expressed as the mean ± SD (n = 5)*,

** *P < 0.01*,

*** *P < 0.001 compared with the infected group*.

*ctrl, the control group; infected: S. japonicum-infected group; pzq-treated: the praziquantel-treated group; me-treated: the myricetin-treated group. neg, negative, indicating that no worms or eggs detected*.

### Myricetin Lessens *S. japonicum*-Induced Pathological Changes in Mouse Liver

**Figure 4:**
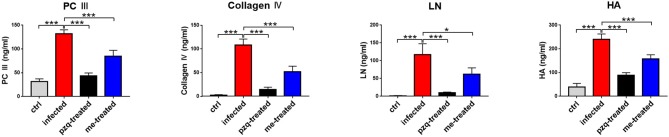
Detection of hepatic fibrosis in *S. japonicum* cercaria infected BALB/c mice with 500 mg/kg praziquantel or 250 mg/kg myricetin for 7days. All mice were randomly divided into four groups: ctrl, the control group; infected: *S. japonicum* infected group; the pzq-treated, the praziquantel-treated group; me-treated, the myricetin-treated group. Levels of type III procollagen (PC III), type IV collagen (collagen IV), laminin (LN) and hyaluronidase (HA) are shown. Data are reported as the mean ± SD (*n* = 3). ^*^P < 0.05, ^***^P < 0.001, compared with the infected group.

**Figure 5:**
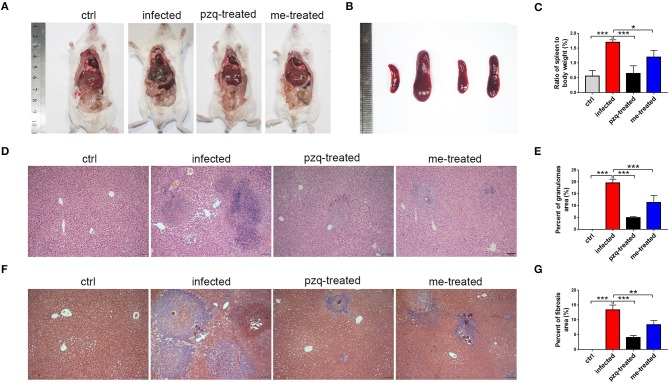
Comparison of the granulomatous inflammation in the liver and pathological changes in the spleen. **(A)** Anatomy images of liver were taken by Nikon DSLR Camera. **(B)** Pathological changes of spleen in different groups (From left to right: ctrl, infected, treated with praziquantel, and treated with myricetin. **(C)** The ratio of spleen to body weight of mice infected with *S. japonicum* cercariae at 7 weeks. **(D)** Representative H&E staining images of hepatic granulomas at 7 weeks post infection with 30 *S. japonicum* cercariae (Scale bars: 100 μm). **(E)** Percent of granulomas in liver were analyzed with Image pro plus 6.0 software. **(F)** Fibrosis changes of liver at 7 weeks post infection with *S. japonicum* were observed by Masson staining (Scale bars: 100 μm). **(G)** The percent of fibrosis area was measured by Image pro plus 6.0. The data are expressed as the mean ± SD (*n* = 3). ^**^P < 0.01, ^***^P < 0.001, compared with the infected group. ctrl, the control group; infected: *S. japonicum* infected group; pzq-treated, the praziquantel-treated group; me-treated, the myricetin-treated group.

The livers of infected mice became gray-black-colored and had a rough surface with numerous irregular whitish micro- and macro-nodules, which were alleviated by myricetin and praziquantel. H&E staining showed that after infection, worm eggs were deposited in the liver surrounded by a large number of inflammatory cells, accompanied by collagen deposition, which formed diffuse egg granulomas. Consistently, ELISA revealed that PC III in mouse serum increased from 31.42 ± 5.58 ng/mL to 131.6 ± 8.75 ng/mL, collagen IV from 2.13 ± 1.47 ng/mL to 108.3 ± 12.58 ng/mL, laminin from 1.13 ± 0.84 ng/mL to 117.3 ± 30.3 ng/mL and hyaluronidase from 39.12 ± 14.89 ng/mL to 239.8 ± 22.89 ng/mL after schistosome infection, indicating obvious liver fibrotic lesions in the mouse liver. An obvious reduction was observed after treatment with myricetin in the serum levels of PC III (84.72 ± 12.32 ng/mL), collagen IV (51.55 ± 12.14 ng/mL), laminin (62.02 ± 17.79 ng/mL) and hyaluronidase (158 ± 16.92 ng/mL) compared to the infected mice [PC III: *F*_(3, 20)_ = 164.3, *P* < 0.001; collagen IV: *F*_(3, 20)_ = 164.4, *P* < 0.001; laminin: *F*_(3, 8)_ = 28, *P* = 0.125and hyaluronidase: *F*_(3, 20)_ = 158.9, *P* < 0.001, respectively], suggesting that myricetin has an inhibitory effect on liver fibrosis caused by *S. japonicum* infection (Figure 4). Furthermore, the size of liver tissue granulomas in the myricetin group was significantly lower vs. the infected group [myricetin: 11.43 ± 2.89% *vs*. infected control: 19.67 ± 1.58%, *F*_(3, 8)_ = 79.74, *P* = 0.0007]. Masson staining further showed that in the infected group, a large amount of collagen fiber was deposited around granulomas and diffusely distributed in the liver tissue, and the size of liver fibrosis area was 13.40 ± 1.64%. Myricetin significantly lessened hepatic fibrosis compared with the control group [8.37 ± 1.40%, *F*_(3, 8)_ = 79.49, *P* = 0.002] (Figure 5).

**Figure 6:**
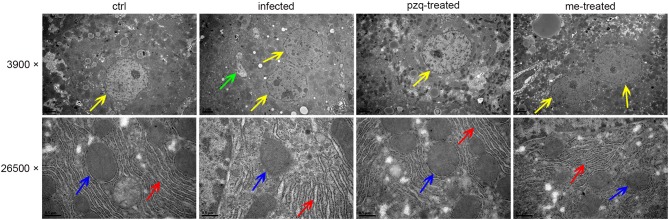
Representative TEM images of pathological changes of liver after treatment of *S. japonicum* infected mice with praziquantel or myricetin. The yellow, green, red and blue arrows indicate cell nucleus, fiber, endoplasmic reticulum and mitochondria, respectively. Scale bars: upper row, 2 μm; lower row, 0.5 μm. ctrl, the control group; infected: *S. japonicum* infected group; the pzq-treated, the praziquantel-treated group; me-treated, the myricetin-treated group.

Additionally, TEM revealed that *S. japonicum* infection could induce cell apoptosis, proliferation of endoplasmic reticulum in hepatocytes, mitochondrial abnormalities such as decreased or absent cristae and membrane disorganization, and formation of fibrous tissue in the mouse liver. In some cases, remarkable hepatocyte apoptosis occurred, showing nuclear shrinkage and pyknosis. The liver pathological damage in infected mice were alleviated after treatment with praziquantel and myricetin (Figure 6).

### Myricetin Shifts Th1/Th2 Balance in *S. japonicum*-Infected Mice

**Figure 7:**
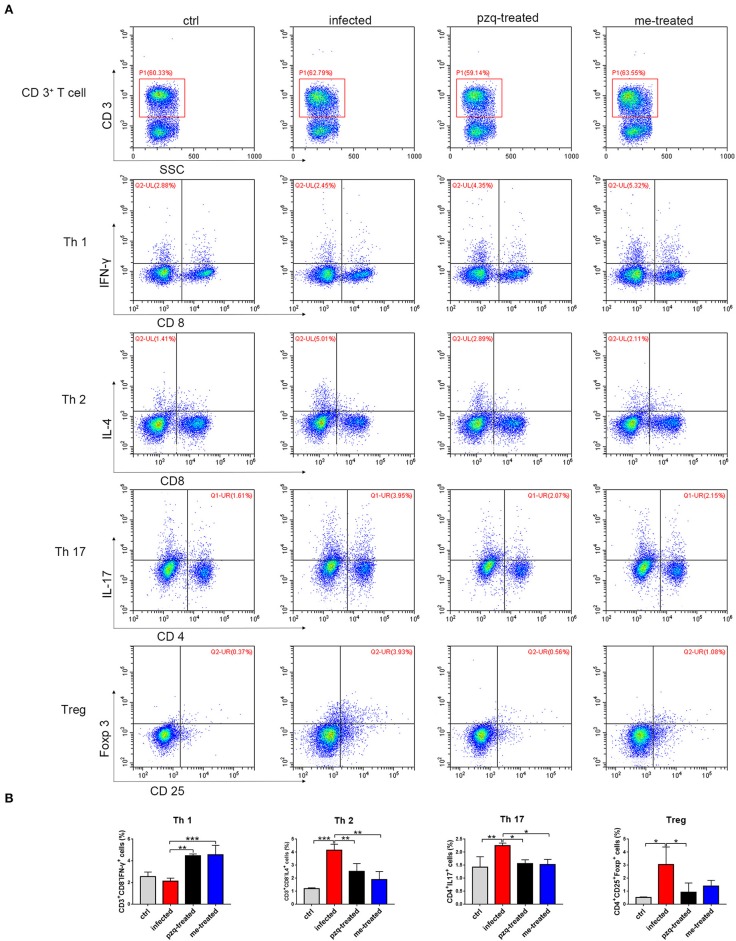
Expression of Th subtypes (Th1/Th2/Th17/Treg) in spleen after treatment of *S. japonicum* infected BALB/c mice with praziquantel or myricetin. **(A)** Flow cytometry results of CD3^+^CD8^−^IFN^−^γ^+^, CD3^+^CD8^−^IL^−^4^+^, CD4^+^IL^−^17^+^ and CD4^+^CD25^+^Foxp^+^ cells in different groups. **(B)** The percentages of CD3^+^CD8^−^IFN^−^γ^+^ (Th1), CD3^+^CD8^−^IL^−^4^+^ (Th2), CD4^+^IL^−^17^+^ (Th17) and CD4^+^CD25^+^Foxp^+^ (Treg) cells were analyzed by flow cytometry. Data were shown as the means ± SD (*n* = 3). ^*^P < 0.05, ^**^P < 0.01, ^***^P < 0.001, compared with the infected group. ctrl, the control group; infected: *S. japonicum* infected group; the pzq-treated, the praziquantel-treated group; me-treated, the myricetin-treated group.

**Figure 8:**
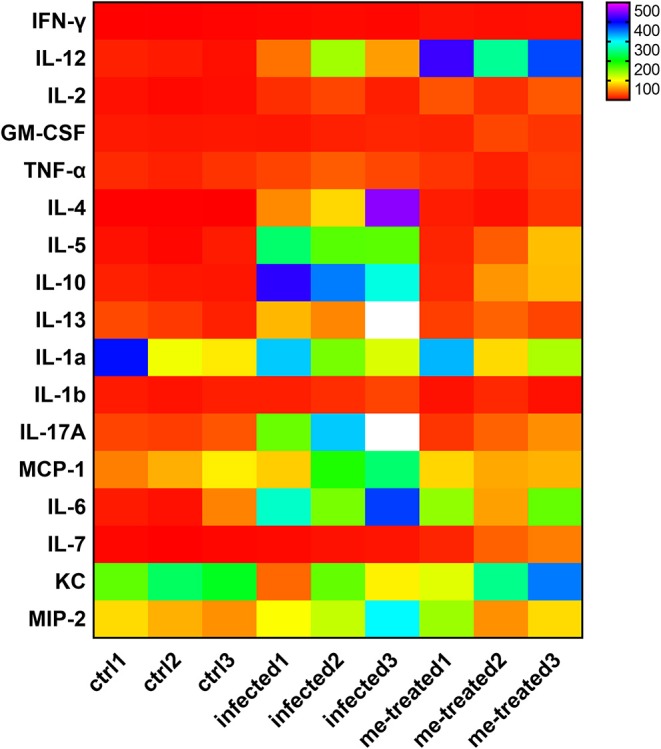
Analysis of serum cytokine changes in *S. japonicum* infected mice after treated with myricetin. The heat map was performed by comparing the serum cytokine levels of different groups, data are representative of three independent experiments. ctrl, the control group; infected: *S. japonicum* infected group; me-treated, the myricetin-treated group.

Flow cytometric analysis of Th subtypes (Th1/Th2/Th17/Treg) in the mouse spleen revealed that *S. japonicum* infection caused no significant change in Th1 cells (2.14 ± 0.274%) in mice, but resulted in an increased in the percentage of Th2 cells from 1.20 ± 0.07% to 4.14 ± 0.46%, and Th17 cells from 1.43 ± 0.40% to 2.25 ± 0.10%, and Treg cells from 0.52 ± 0.051% to 3.05 ± 1.34%. Myricetin significantly up-regulated Th1 cells in infected mice [4.56 ± 0.87%, *F*_(3, 8)_ = 18.81, *P* < 0.001] and reduced the proportion of Th2 cells [1.89 ± 0.62%, *F*_(3, 8)_ = 19.65, *P* = 0.001] and Th17 cells [1.527 ± 0.1973%, *F*_(3, 8)_ = 7.398, *P* = 0.015]. In addition, myricetin did not significantly reduced the proportion of Treg in infected mice (1.39 ± 0.44%) (*P* > 0.05) (Figure 7). We further examined whether myricetin induced changes in serum cytokines in *S. japonicum*-infected mice. ELISA showed that *S. japonicum* infection caused apparent increase in the plasma levels of IL-12, IL-2, TNF-α, IL-4, IL-5, IL-10, IL-13, IL-17A, IL-6, and MIP-2. Myricetin attenuated the rise in the plasma levels of IL-4, IL-5, IL-10, IL-13, and IL-17A in infected mice while increasing the plasma contents of IFN-γ, IL-12, and IL-7 (Figure 8).

### Myricetin Attenuates *S. japonicum*-Induced Liver Fibrosis by Regulating TGFβ1/Smad/ERK Signaling

**Figure 9:**
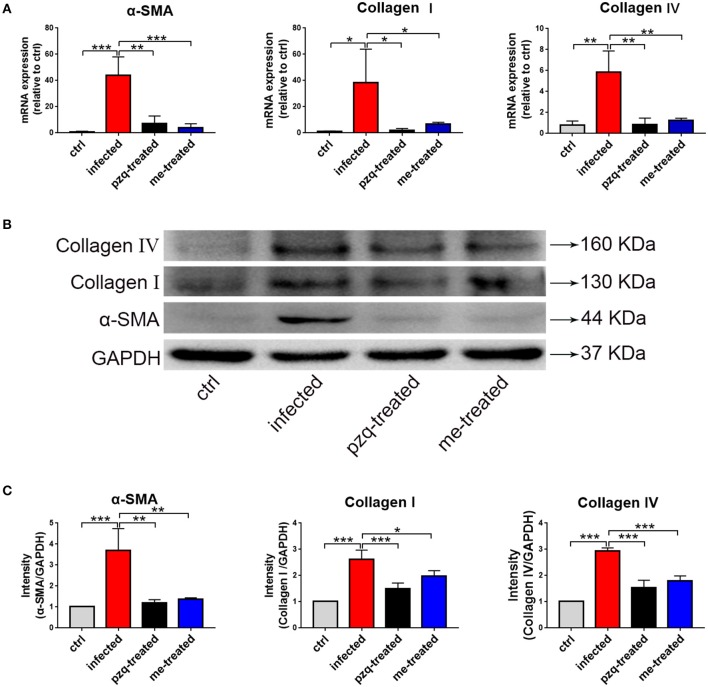
RT-qPCR and western blot analyses of alpha-smooth muscle actin (α-SMA), Collagen I and Collagen IV in liver of *S. japonicum* infected mice after treated with praziquantel or myricetin. **(A)** RT-qPCR products obtained with the total RNA of liver with α-SMA-, Collagen I- or Collagen IV-specific primers. **(B)** The expression levels of α-SMA, Collagen I and Collagen IV in liver were confirmed by western blot. **(C)** Densitometric analysis of α-SMA, Collagen I and Collagen IV normalized to the endogenous control (GAPDH) and expressed as fold change. Data are expressed as the mean ± SD (n=3). ^*^P < 0.05, ^**^P < 0.01, ^***^P < 0.001, compared with the infected group. ctrl, the control group; infected: *S. japonicum* infected group; the pzq-treated, the praziquantel-treated group; me-treated, the myricetin-treated group.

We further examined the expression of α-SMA, collagen I and IV in liver tissue. Our RT-PCR assays showed that myricetin caused a 10-, 6-, and 4.6-fold reduction in the mRNA transcript levels of α-SMA, collagen I and IV in infected mice compared to non-treated infected mice [*F*_(3, 8)_ = 19.81, *P* < 0.001]; *F*_(3, 8)_ = 5.63, *P* = 0.04 and *F*_(3, 8)_ = 15.5, *P* = 0.002, respectively) (Figure 9A). Consistent findings were also found in Western blotting assays [α-SMA: *F*_(3, 8)_ = 19.81, *P* = 0.002; collagen I: *F*_(3, 8)_ = 27.44, *P* = 0.022; collagen IV: *F*_(3, 8)_ = 63.82, *P* < 0.001] (Figures 9B,C), indicating that myricetin can effectively inhibit schistosome-induced upregulation of hepatic fibrosis-related proteins in mice.

**Figure 10:**
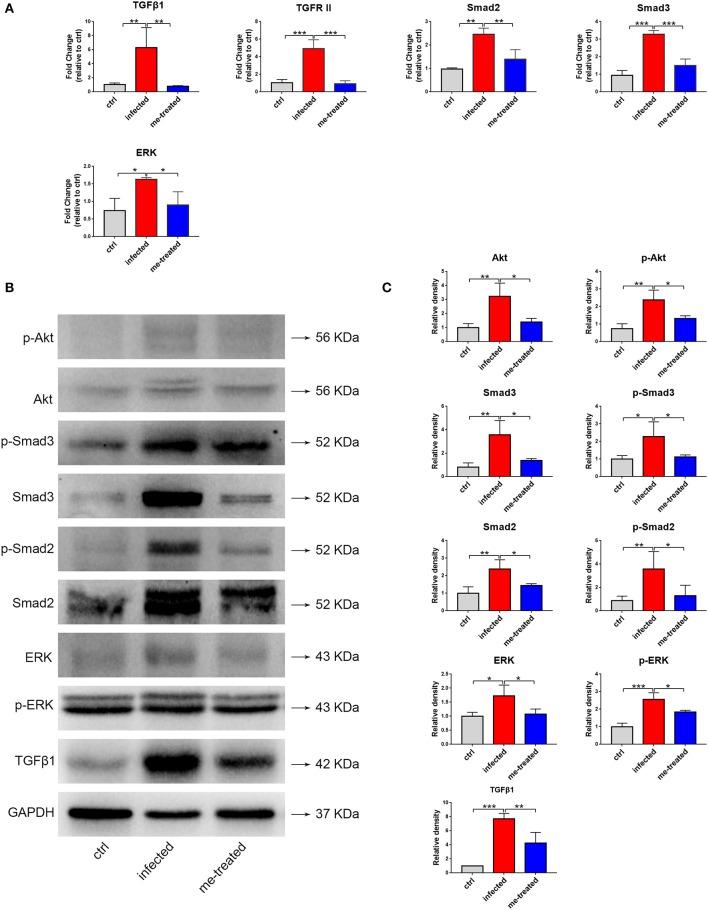
Effects of myricetin on the TGF-β1-induced signaling pathway in *S. japonicum* infected mice model. **(A)** The mRNA expression of TGFβ1, TGFR II, smad2, smad3 and ERK was detected by RT-qPCR. **(B)** Western blot analysis for TGFβ1, ERK, p-ERK, Smad2, p-Smad2, Smad3, p-Smad3, Akt and p-Akt. **(C)** The relative densitometry of TGFβ1, ERK, p-ERK, Smad2, p-Smad2, Smad3, p-Smad3, Akt and p-Akt (relative to the endogenous control, GAPDH) and expressed as fold change. The values are expressed as the mean ± SD. ^*^P < 0.05, ^**^P < 0.01, ^***^P < 0.001 compared with the infected group. ctrl, the control group; infected: *S. japonicum* infected group; me-treated, the myricetin-treated group.

TGFβ1 signaling is important in activating hepatic stellate cells and implicated in hepatic fibrosis. Our RT-qPCR assays showed that schistosome infection caused a 6.3-, 4.9-, 2.5-, 3.5-, and 2.2-fold increase in the mRNA transcript levels of TGFβ1, TGFRII, Smad2, Smad3, and ERK in mouse liver tissues. Meanwhile, myricetin effectively suppressed schistosome-induced upregulation of the mRNA transcript levels of TGFβ1 [*F*_(3, 8)_ = 10.86, *P* = 0.004], TGFR II [*F*_(2, 6)_ = 36.32, *P* < 0.001], Smad2 [*F*_(2, 6)_ = 21.74, *P* = 0.007], Smad3 [*F*_(2, 6)_ = 51.01, *P* < 0.001], and ERK1/2 [*F*_(2, 6)_ = 7.719, *P* = 0.041] in the liver tissues of infected mice. Western blotting assays further showed that myricetin effectively inhibited the protein expression of TGFβ1 [*F*_(2, 6)_ = 33.9, *P* = 0.009], ERK [*F*_(2, 6)_ = 6.797, *P* = 0.041], phospho-ERK [*F*_(2, 6)_ = 28.58, *P* = 0.022], Smad2 [*F*_(2, 6)_ = 10.13, *P* = 0.044], phospho-Smad2 [*F*_(2, 6)_ = 7.862, *P* = 0.026], Smad3 [*F*_(2, 6)_ = 11.78, *P* = 0.019], p-Smad3 [*F*_(2, 6)_ = 6.107, *P* = 0.050], Akt [*F*_(2, 6)_ = 12.37, *P* = 0.015], phospho-Akt [*F*_(2, 6)_ = 15.32, *P* = 0.023] in the liver tissues of infected mice (Figure 10). Thus, we inferred that myricetin may reduce liver fibrosis induced by *S. japonicum* infection by regulating the TGFβ1/Smad/ERK and PI3K/Akt signaling pathway.

## Discussion

Praziquantel has been widely used as an anti-schistosome drug, which was hailed as a breakthrough in the chemotherapy of schistosomiasis and has greatly promoted the prevention and control of schistosomiasis. However, whether repeated uses of praziquantel for large-scale chemotherapy would lead to the occurrence of resistance has received widespread attention. For *S. japonicum*, no evidence shows reduced sensitivity to praziquantel (12), but long-term reliance on a drug may have the potential risk of resistance. In this study, large-scale screening of the small-molecule compound library revealed that myricetin has a good helminthicidal effect on *S. japonicum in vitro*. We demonstrated that myricetin attenuated liver fibrosis in mice *via* modulating TGFβ1 and Akt signaling and shifting Th1/Th2 balance. Our study provides the first piece of direct experimental evidence that myricetin possesses potent anti-schistosome activities *in vitro* and *in vivo* and offers new insights into the mechanisms of action by myricetin, indicating that myricetin could be further explored as a therapeutic agent for *S. japonica*.

Myricetin is a common natural flavonol compound which can be detected in *Myrica rubra, Vaccinium macrocarponl Ait, Ribes nigrum L., Vaccinium uliginosum Linn*. and *Semen Trigonellae* (24). Myricetin possesses a variety of pharmacological activities such as anti-oxidation, anti-tumor, anti-inflammatory, anti-microbial, anti-allergy, protection of cardiovascular and neurons (25–27) and has little toxic side effects (28). In addition, myricetin possesses hepatoprotective effects (29). At present, the effects of myricetin on parasites and hosts have not been reported yet. In this paper, we found that myricetin has good anti-*S. japonicum* activity. The findings of *in vitro* experiments illustrated that with the prolongation of the incubation time and the increase of drug concentration, the mortality of adult *S. japonicum* was increased and the activity of the worms decreased, indicating that the effect of myricetin on *S. japonicum* adult worms was time- and concentration-dependent. The entire worm is surrounded by a continuous cytoplasmic membrane, or syncytium, known as the tegument, composed of surface membrane, matrix and basal membrane, which is a unique structure of all trematodes. This structure is closely related to immune escape, nutrient uptake, excretion of catabolites, targeted drug absorption and other physiological processes (30–32), and most effective drugs against schistosomes would damage the tegument, including praziquantel (33), oxamycin (34) and artemisinin (35). After treatment with myricetin, *S. japonicum*'s oral and abdominal suckers, sensory sensory papillae and on the surface, tegument and the reproductive organs were damaged to some extent, which may be due to the disruption of the osmotic pressure balance of the worm body, leading to changes in the body structure of the worm and eventually death.

Apoptosis is a normal process in growth and development of many organisms. Excessive activation or inhibition of apoptotic signals may result in the occurrence and development of many diseases, especially cancer (36). The levels of apoptosis of *S. japonicum* adult worms in different definite hosts are different, and are significantly higher in rats than mice (37). Under TEM, typical apoptotic phenomena were seen in both male and female worms treated with myricetin, including cell shrinkage, and pyknosis, but no mitochondrial damage was observed. The caspase family, the Bcl-2 family, cytokine-induced apoptosis inhibitor and apoptosis inhibitory factor (IAP) are involved in the apoptosis of *S. japonicum* (38–40). Our RT-qPCR assays showed that the expression of caspase 7, CYC and APAF in males and Bak, CYC, caspase 7, APAF, AIF and API in females elevated, while the anti-apoptotic gene bcl-2 decreased in both sexes, suggesting that the reduction of the worm's vitality and even death may be due to the drug *via* modulating the transcription level of the apoptotic genes of the worm. Additionally, flow cytometry revealed that the percentage of apoptotic cells in males was significantly higher than that in females after the action of myricetin, which is probably due to differences in the tegument structure and function between the male and female worms. The males are more sensitive to the stimulation of the surrounding environment with a loose tegument. In summary, *in vitro* results showed that myricetin had a good helminthicidal effect, and the damage to the tegument and the occurrence of worm cell apoptosis provide a basis for myricetin's helminthicidal action mechanism.

In the animal model of schistosome infection, Th1 type immune response is dominated in the acute phase, and produce cytokines such as IFN-γ, IL-12, and TNF-α, which can inhibit the invasion of pathogens by killing them (41). Th1 type immune response also has a certain inhibitory effect on granuloma and liver fibrosis. After 4 weeks of infection, the host's immune response shifts to a Th2 type, producing cytokines such as IL-4, IL-10, and IL-13. As the infection enters the chronic phase, the egg granuloma size becomes smaller, and Treg cells are activated, and Th1/Th2 immune balance is regulated and maintained, thereby inhibiting the development of liver fibrotic lesions (42, 43). Additionally, Th17 cells are positively correlated with liver pathological damage, and the use of IL-17 neutralizing antibodies can effectively lessen egg granulomatous lesions (44). By detecting CD4^+^ T cell subtypes from spleen cells of mice infected with *S. japonicum* after 7 weeks of infection, we found that myricetin increased the proportion of Th1 cells but decreased the proportion of Th2 and Th17 cells, and ELISA also demonstrated elevations in plasma IFN-γ, IL-12, and IL-7 with concurrent reductions in plasma IL-4, IL-5, IL-10, IL-13, and IL-17A, indicating that myricetin modulates the immune response in schistosome-infected mice. Furthermore, we found that myricetin significantly reduced the area of liver granulomas and alleviated liver fibrosis. Egg granuloma is the basis of development of liver fibrosis induced by schistosome infection. Soluble egg antigens stimulate the production by macrophages and lymphocytes of various cytokines such as PDGF and TGFβ1 to induce the activation and proliferation of hepatic stellate cells (HSCs), which then transform into myofibroblasts capable of producing collagen. The activation of HSC is key to the formation of liver fibrosis (45). Activation of HSCs leads to increased expression of profibrotic factors such as α-SMA, collagen I and IV, cytoplasmic expansion, excessive synthesis and less degradation of ECM, leading to fibrosis. The main components of the ECM include hyaluronidase, PC III, collagen IV and laminin, which are commonly used as indicators of liver fibrosis clinically (46). Myricetin effectively inhibited *S. japonicum*-induced upregulation of liver fibrosis factor α-SMA, collagen I and IV and plasma hyaluronidase, PC III, collagen IV and laminin in mice, indicating that myricetin significantly lessened liver fibrosis in mice.

TGFβ1 signaling is the main regulatory mechanism in liver fibrosis. In TGFβ1-Smad signaling, TGFβ1 first activates intracellular signals by binding to TGFβ II, and then TGFβ1 activates TGF-β receptor type I (TβRI) kinase, resulting in phosphorylation of Smad2 and Smad3. Subsequently, activated Smad2 and Smad3 form a hetero-oligomer with Smad4, and then the Smad complex translocates to the nucleus, where it regulates transcription of target genes (47, 48). TGFβ1-Smad continues to activate ERK1/2 signaling (49, 50). TGFβ1/Smad/ERK signaling can promote liver fibrosis (51). In addition, TGFβ1 has been reported to activate PI3K/Akt signaling (52). Activation of Akt can not only promote the proliferation and migration of HSCs, but also promote the production of ECM by HSCs (53). Our study showed that myricetin suppressed the expression of TGFβ1, phospho-Smad2, phospho-Smad3, phosph-ERK1/2, Akt, phospho-Akt in schistosome-infected mice, revealing that myricetin may reduce liver fibrosis in schistosome-infected mice by inhibiting TGFβ1/Smad/ERK and PI3K/Akt signaling.

In conclusion, our study provides evidence for the first time that myricetin has anti-*S. japonicum* effects *in vitro* and *in vivo*. Meanwhile, myricetin has a significant effect on reducing liver fibrosis in schistosome-infected hosts, suggesting that myricetin with low toxicity may be explored as a novel therapeutic drug against *S. japonicum*.

## Data Availability Statement

All datasets generated for this study are included in the article/Supplementary Files.

## Ethics Statement

The animal study was reviewed and approved by The Institutional Animal Care and Use Committee of Sun Yat-sen University.

## Author Contributions

ZL conceived and designed the study. ZL and PH drafted the manuscript. PH, MZ, and SC carried out the experiments. YH, MG, YM, and YL participated in data analysis. HZ, PD, and YC participated in study design, technological guidance and coordination. The final manuscript was read and approved by all authors.

### Conflict of Interest

The authors declare that the research was conducted in the absence of any commercial or financial relationships that could be construed as a potential conflict of interest.
