# Supplementary material for: Myricetin Possesses Anthelmintic Activity and Attenuates Hepatic Fibrosis via Modulating TGFβ1 and Akt Signaling and Shifting Th1/Th2 Balance in Schistosoma japonicum-Infected Mice
Source: Front Immunol. 2020 Apr 16;11:593. doi: 10.3389/fimmu.2020.00593 (PMC7176910; doi:10.3389/fimmu.2020.00593)
Supplement: Supplementary file 1 [file Table_1.docx]

Supplemental Table 1. Large-scale screening of drugs with potential insecticidal effects on *Schistosoma japonicum*. Pairs of worms were incubated with 1000 μM concentrations of different drugs and their viability was scored at 24, 48 and 72 h, respectively, using a viability scale of 0–3 (3 = normally active; 2 = slowed activity; 1 = minimal activity, occasional movement of head and tail; 0 =total absence of mobility). ctrl: adult worms were incubated in RPMI 1640 medium with 1% DMSO. Pzq: praziquantel (100 μM) was used as positive control groups. VDR: Vitality Decrease Rate.

| Drugs  (C: 1000 μmol/L） | pairs of worms | 24 h | 48 h | 72 h |
| --- | --- | --- | --- | --- |
|  |  | score/VDR（%） | score/VDR（%） | score/VDR（%） |
| ctrl | 3 | 18.0±0.0/0.0 | 18.0±0.0/0.0 | 18.0±0.0/0.0 |
| praziquantel | 3 | 0.0±0.0/100.0 | 0.0±0.0/100.0 | 0.0±0.0/100.0 |
| propofol | 3 | 18.0±0.0/0.0 | 18.0±0.0/0.0 | 18.0±0.0/0.0 |
| etofenamate | 3 | 18.0±0.0/0.0 | 17.0±1.0/6.0 | 16.7±1.2/7.0 |
| almotriptan malate | 3 | 17.0±1.0/6.0 | 16.3±0.6/9.0 | 16.3±0.6/9.0 |
| betamethasone | 3 | 18.0±0.0/0.0 | 15.3±0.6/15.0 | 14.7±0.6/19.0 |
| ala hydrochloride | 3 | 18.0±0.0/0.0 | 18.0±0.0/0.0 | 16.7±1.2/7.0 |
| liothyronine sodium | 3 | 18.0±0.0/0.0 | 16.7±1.2/7.0 | 15.3±0.6/15.0 |
| lupetidin | 3 | 18.0±0.0/0.0 | 18.0±0.0/0.0 | 16.7±1.2/7.0 |
| amidrine | 3 | 18.0±0.0/0.0 | 16.7±1.5/7.0 | 16.7±1.5/7.0 |
| **myricetin** | 3 | **0.0±0.0/100.0** | **0.0±0.0/100.0** | **0.0±0.0/100.0** |
| fenipentol | 3 | 18.0±0.0/0.0 | 17.3±0.6/4.0 | 17.0±1.0/6.0 |
| benzyl alcohol | 3 | 18.0±0.0/0.0 | 18.0±0.0/0.0 | 16.7±1.2/7.0 |
| roniacol | 3 | 16.0±1.0/11.0 | 15.7±0.6/13.0 | 15.3±0.6/15.0 |
| pargyline | 3 | 18.0±0.0/0.0 | 16.7±1.2/7.0 | 12.7±1.2/29.4 |
| triacetin | 3 | 17.0±0.0/6.0 | 16.0±0.0/11.1 | 15.7±0.6/13.0 |
| cis-anethol | 3 | 18.0±0.0/0.0 | 18.0±0.0/0.0 | 18.0±0.0/0.0 |
| birch-me | 3 | 18.0±0.0/0.0 | 18.0±0.0/0.0 | 18.0±0.0/0.0 |
| benzyl benzoate | 3 | 17.7±0.6/2.0 | 17.7±0.6/2.0 | 15.3±0.6/15.0 |
| retinyl acetate | 3 | 18.0±0.0/0.0 | 18.0±0.0/0.0 | 16.7±1.2/7.0 |
| cresol | 3 | 18.0±0.0/0.0 | 18.0±0.0/0.0 | 17.3±0.6/4.0 |
| anestan | 3 | 17.3±0.6/4.0 | 17.0±0.0/6.0 | 16.0±1.0/11.0 |
| resorcin monoacetate | 3 | 18.0±0.0/0.0 | 18.0±0.0/0.0 | 18.0±0.0/0.0 |
| itopride hydrochloride | 3 | 18.0±0.0/0.0 | 17.0±1.0/6.0 | 15.67±1.5/13.0 |
| acetylcholine chloride | 3 | 17.0±1.0/6.0 | 16.3±0.6/9.0 | 15.3±1.2/15.0 |
| ergonovine maleate | 3 | 18.0±0.0/0.0 | 18.0±0.0/0.0 | 18.0±0.0/0.0 |
| modafinil | 3 | 18.0±0.0/0.0 | 18.0±0.0/0.0 | 17.7±0.6/2.0 |
| sunitinib malate | 3 | 18.0±0.0/0.0 | 16.7±1.2/7.0 | 15.3±0.6/15.0 |
| pheniramine maleate | 3 | 18.0±0.0/0.0 | 17.0±0.0/6.0 | 16.7±0.6/7.0 |
| sulindac | 3 | 18.0±0.0/0.0 | 16.7±1.5/7.0 | 16.7±1.5/7.0 |
| sulfacetamide sodium | 3 | 18.0±0.0/0.0 | 17.3±0.6/4.0 | 17.0±1.0/6.0 |
| antepan | 3 | 18.0±0.0/0.0 | 18.0±0.0/0.0 | 16.7±1.2/7.0 |
| hydroxyquinoline | 3 | 16.0±1.0/11.0 | 15.7±0.6/13.0 | 13.0±1.0/28.0 |
| thioridazine hydrochloride | 3 | 18.0±0.0/0.0 | 16.7±1.2/7.0 | 14.3±0.6/20.0 |
| clozapine | 3 | 17.0±0.0/6.0 | 16.0±0.0/11.1 | 15.7±0.6/13.0 |
| phenytoin sodium | 3 | 18.0±0.0/0.0 | 18.0±0.0/0.0 | 18.0±0.0/0.0 |
| oxethazaine | 3 | 18.0±0.0/0.0 | 18.0±0.0/0.0 | 18.0±0.0/0.0 |
| cortisone acetate | 3 | 17.7±0.6/2.0 | 16.3±0.6/9.0 | 15.3±0.6/15.0 |
| betaine hydrochloride | 3 | 18.0±0.0/0.0 | 18.0±0.0/0.0 | 16.7±1.2/7.0 |
| famprofazone | 3 | 18.0±0.0/0.0 | 18.0±0.0/0.0 | 17.3±0.6/4.0 |
| acetaminophen | 3 | 18.0±0.0/0.0 | 18.0±0.0/0.0 | 16.0±1.7/11.0 |
| halcinonide | 3 | 18.0±0.0/0.0 | 18.0±0.0/0.0 | 18.0±0.0/0.0 |
| trichlormethiazide | 3 | 18.0±0.0/0.0 | 16.0±1.0/11.0 | 14.0±2.0/22.0 |
| (±)-bisoprolol hemifumarate | 3 | 18.0±0.0/0.0 | 18.0±0.0/0.0 | 17.0±1.0/6.0 |
| mitoxantrone hydrochloride | 3 | 18.0±0.0/0.0 | 18.0±0.0/0.0 | 18.0±0.0/0.0 |
| maprotiline hydrochloride | 3 | 18.0±0.0/0.0 | 17.0±0.0/6.0 | 16.0±1.7/11.0 |
| escitalopram | 3 | 18.0±0.0/0.0 | 18.0±0.0/0.0 | 18.0±0.0/0.0 |
| terazosin hydrochloride | 3 | 18.0±0.0/0.0 | 18.0±0.0/0.0 | 17.3±0.6/4.0 |
| azelastine | 3 | 18.0±0.0/0.0 | 18.0±0.0/0.0 | 18.0±0.0/0.0 |
| pizotifen malate | 3 | 16.0±1.0/11.0 | 15.3±0.6/15.0 | 14.0±1.0/22.0 |
| trimetozine | 3 | 18.0±0.0/0.0 | 18.0±0.0/0.0 | 16.7±1.2/7.0 |
| gemcitabine | 3 | 18.0±0.0/0.0 | 16.7±1.2/7.0 | 15.3±0.6/15.0 |
| zonisamide | 3 | 18.0±0.0/0.0 | 18.0±0.0/0.0 | 18.0±0.0/0.0 |
| spiperone | 3 | 18.0±0.0/0.0 | 16.7±1.5/7.0 | 16.7±1.5/7.0 |
| clofarabine | 3 | 18.0±0.0/0.0 | 17.3±0.6/4.0 | 17.0±1.0/6.0 |
| suxibuzone | 3 | 18.0±0.0/0.0 | 18.0±0.0/0.0 | 16.7±1.2/7.0 |
| d-sorbitol | 3 | 16.0±1.0/11.0 | 15.7±0.6/13.0 | 13.0±1.0/28.0 |
| labetalol hydrochloride | 3 | 18.0±0.0/0.0 | 16.7±1.2/7.0 | 12.7±1.2/30.0 |
| cefadroxil | 3 | 17.0±0.0/6.0 | 16.0±0.0/11.1 | 15.7±0.6/13.0 |
| erythromycin ethylsuccinate | 3 | 18.0±0.0/0.0 | 18.0±0.0/0.0 | 18.0±0.0/0.0 |
| hexetidine | 3 | 18.0±0.0/0.0 | 18.0±0.0/0.0 | 18.0±0.0/0.0 |
| miconazole nitrate | 3 | 18.0±0.0/0.0 | 17.3±0.6/4.0 | 17.0±1.0/6.0 |
| clofibrate | 3 | 18.0±0.0/0.0 | 18.0±0.0/0.0 | 18.0±0.0/0.0 |
| arsenic oxide (3) | 3 | 18.0±0.0/0.0 | 15.7±0.6/13.0 | 12.3±0.6/31.0 |
| zolpidem | 3 | 18.0±0.0/0.0 | 16.7±1.2/7.0 | 15.3±0.6/15.0 |
| ethaverine hydrochloride | 3 | 17.0±0.0/6.0 | 16.0±0.0/11.1 | 15.7±0.6/13.0 |
| indometacin | 3 | 18.0±0.0/0.0 | 18.0±0.0/0.0 | 18.0±0.0/0.0 |
| fluoxetine | 3 | 18.0±0.0/0.0 | 18.0±0.0/0.0 | 18.0±0.0/0.0 |
| inositol | 3 | 18.0±0.0/0.0 | 17.7±0.6/2.0 | 15.3±0.6/15.0 |
| retigabine | 3 | 18.0±0.0/0.0 | 18.0±0.0/0.0 | 16.7±1.2/7.0 |
| moxisylyte hydrochloride | 3 | 18.0±0.0/0.0 | 18.0±0.0/0.0 | 18.0±0.0/0.0 |
| cilostazol | 3 | 18.0±0.0/0.0 | 18.0±0.0/0.0 | 16.0±1.7/11.0 |
| naphazoline hydrochloride | 3 | 18.0±0.0/0.0 | 18.0±0.0/0.0 | 18.0±0.0/0.0 |
| atenolol | 3 | 18.0±0.0/0.0 | 17.0±1.0/6.0 | 16.3±0.6/9.0 |
| adiphenine hydrochloride | 3 | 18.0±0.0/0.0 | 18.0±0.0/0.0 | 18.0±0.0/0.0 |
| miltefosine | 3 | 18.0±0.0/0.0 | 18.0±0.0/0.0 | 17.3±0.6/4.0 |
| sodium 4-aminosalicylate | 3 | 18.0±0.0/0.0 | 17.0±0.0/6.0 | 16.0±1.7/11.0 |
| anovigam | 3 | 18.0±0.0/0.0 | 18.0±0.0/0.0 | 18.0±0.0/0.0 |
| urapidil hydrochloride | 3 | 18.0±0.0/0.0 | 17.0±1.0/6.0 | 16.7±1.2/7.0 |
| bumetanide | 3 | 18.0±0.0/0.0 | 16.3±0.6/9.0 | 16.3±0.6/9.0 |
| glimepiride | 3 | 18.0±0.0/0.0 | 18.0±0.0/0.0 | 18.0±0.0/0.0 |
| felodipine | 3 | 18.0±0.0/0.0 | 18.0±0.0/0.0 | 16.7±1.2/7.0 |
| pranoprofen | 3 | 18.0±0.0/0.0 | 16.7±1.2/7.0 | 15.3±0.6/15.0 |
| nylidrin hydrochloride | 3 | 18.0±0.0/0.0 | 18.0±0.0/0.0 | 16.7±1.2/7.0 |
| amorolfine hydrochloride | 3 | 18.0±0.0/0.0 | 16.7±1.5/7.0 | 16.7±1.5/7.0 |
| cephradine | 3 | 18.0±0.0/0.0 | 17.3±0.6/4.0 | 17.0±1.0/6.0 |
| sumatriptan succinate | 3 | 18.0±0.0/0.0 | 18.0±0.0/0.0 | 16.7±1.2/7.0 |
| betaxolol hydrochloride | 3 | 18.0±0.0/0.0 | 15.7±0.6/13.0 | 15.3±0.6/15.0 |
| sulfanilylurea | 3 | 18.0±0.0/0.0 | 16.7±1.2/7.0 | 16.7±1.2/7.0 |
| methscopolamine | 3 | 17.0±0.0/6.0 | 17.0±0.0/6.0 | 15.7±0.6/13.0 |
| lobeline hydrochloride | 3 | 18.0±0.0/0.0 | 18.0±0.0/0.0 | 18.0±0.0/0.0 |
| trimethadione | 3 | 18.0±0.0/0.0 | 17.0±1.0/6.0 | 16.7±1.2/7.0 |
| dimenhydrinate | 3 | 17.0±1.0/6.0 | 17.0±1.0/6.0 | 16.3±0.6/9.0 |
| colchicine | 3 | 18.0±0.0/0.0 | 15.3±0.6/15.0 | 14.7±0.6/19.0 |
| aniracetam | 3 | 18.0±0.0/0.0 | 18.0±0.0/0.0 | 16.7±1.2/7.0 |
| riluzole | 3 | 18.0±0.0/0.0 | 18.0±0.0/0.0 | 18.0±0.0/0.0 |
| fluocinolone acetonide | 3 | 18.0±0.0/0.0 | 18.0±0.0/0.0 | 18.0±0.0/0.0 |
| sertraline hydrochloride | 3 | 18.0±0.0/0.0 | 18.0±0.0/0.0 | 16.7±1.5/7.0 |
| ozagrel hydrochloride | 3 | 18.0±0.0/0.0 | 17.3±0.6/4.0 | 17.0±1.0/6.0 |
| betahistine 2hydrochloride | 3 | 18.0±0.0/0.0 | 18.0±0.0/0.0 | 16.7±1.2/7.0 |
| dimethyl sulfoxide | 3 | 18.0±0.0/0.0 | 18.0±0.0/0.0 | 15.3±0.6/15.0 |
| oritavancin diphosphate | 3 | 18.0±0.0/0.0 | 16.7±1.2/7.0 | 15.7±0.6/13.0 |
| cinchophen | 3 | 18.0±0.0/0.0 | 16.0±0.0/11.1 | 15.7±0.6/13.0 |
| probenecid | 3 | 18.0±0.0/0.0 | 18.0±0.0/0.0 | 18.0±0.0/0.0 |
| menadione | 3 | 18.0±0.0/0.0 | 17.3±0.6/4.0 | 16.7±1.2/7.0 |
| oxcarbazepine | 3 | 18.0±0.0/0.0 | 16.3±0.6/9.0 | 16.3±0.6/9.0 |
| pamabrom | 3 | 18.0±0.0/0.0 | 18.0±0.0/0.0 | 18.0±0.0/0.0 |
| amodiaquin dihydrochloride dihydrate | 3 | 18.0±0.0/0.0 | 18.0±0.0/0.0 | 17.3±0.6/4.0 |
| dibucaine | 3 | 18.0±0.0/0.0 | 16.7±1.2/7.0 | 15.3±0.6/15.0 |
| promethazine | 3 | 18.0±0.0/0.0 | 18.0±0.0/0.0 | 17.0±1.0/7.0 |
| homatropine methylbromide | 3 | 18.0±0.0/0.0 | 18.0±0.0/0.0 | 17.3±0.6/4.0 |
| methylene blue trihydrate | 3 | 18.0±0.0/0.0 | 18.0±0.0/0.0 | 18.0±0.0/0.0 |
| xylometazoline hydrochloride | 3 | 18.0±0.0/0.0 | 18.0±0.0/0.0 | 16.7±1.2/7.0 |
| dl-xylose | 3 | 16.0±1.0/11.0 | 15.7±0.6/13.0 | 13.0±1.0/28.0 |
| vanatone | 3 | 18.0±0.0/0.0 | 16.7±1.2/7.0 | 15.3±0.6/15.0 |
| diphenylpyraline hydrochloride | 3 | 18.0±0.0/0.0 | 16.0±0.0/11.1 | 15.7±0.6/13.0 |
| cetirizine 2hydrochloride | 3 | 18.0±0.0/0.0 | 18.0±0.0/0.0 | 18.0±0.0/0.0 |
| molsidomine | 3 | 18.0±0.0/0.0 | 17.0±1.0/6.0 | 16.7±1.2/7.0 |
| sanguinarine chloride | 3 | 17.0±1.0/6.0 | 16.3±0.6/9.0 | 15.3±0.6/15.0 |
| vagistat | 3 | 18.0±0.0/0.0 | 18.0±0.0/0.0 | 18.0±0.0/0.0 |
| anagrelide | 3 | 18.0±0.0/0.0 | 18.0±0.0/0.0 | 18.0±0.0/0.0 |
| amiloride hydrochloride | 3 | 18.0±0.0/0.0 | 16.7±1.2/7.0 | 15.3±0.6/15.0 |
| fluticasone propionate | 3 | 18.0±0.0/0.0 | 18.0±0.0/0.0 | 16.7±1.2/7.0 |
| thioctic acid | 3 | 18.0±0.0/0.0 | 17.3±0.6/4.0 | 16.7±1.5/7.0 |
| seratrodast | 3 | 18.0±0.0/0.0 | 18.0±0.0/0.0 | 17.0±1.0/6.0 |
| bromisoval | 3 | 18.0±0.0/0.0 | 18.0±0.0/0.0 | 16.7±1.2/7.0 |
| tilorone 2hydrochloride | 3 | 16.0±1.0/11.0 | 13.3±1.2/26.0 | 11.7±2,5/35.0 |
| probucol | 3 | 18.0±0.0/0.0 | 18.0±0.0/0.0 | 18.0±0.0/0.0 |
| meclofenamate sodium | 3 | 17.0±0.0/6.0 | 16.0±0.0/11.1 | 12.3±3.5/31.0 |
| sulfogaiacol | 3 | 18.0±0.0/0.0 | 18.0±0.0/0.0 | 18.0±0.0/0.0 |
| lactitol monohydrate | 3 | 18.0±0.0/0.0 | 17.0±1.0/6.0 | 16.7±1.2/7.0 |
| fipexide | 3 | 17.0±1.0/6.0 | 16.3±0.6/9.0 | 15.0±0.0/17.0 |
| rosiglitazone | 3 | 18.0±0.0/0.0 | 18.0±0.0/0.0 | 18.0±0.0/0.0 |
| pregabalin | 3 | 18.0±0.0/0.0 | 18.0±0.0/0.0 | 16.7±1.2/7.0 |
| cinepazide maleate | 3 | 18.0±0.0/0.0 | 16.7±1.2/7.0 | 15.3±0.6/15.0 |
| sotalol hydrochloride | 3 | 18.0±0.0/0.0 | 18.0±0.0/0.0 | 16.7±1.2/7.0 |
| 1,2-diphenylhydrazine | 3 | 18.0±0.0/0.0 | 18.0±0.0/0.0 | 18.0±0.0/0.0 |
| dihydroergotamine mesylate | 3 | 18.0±0.0/0.0 | 17.3±0.6/4.0 | 16.3±1.2/9.0 |
| 4-methylpyrazole | 3 | 18.0±0.0/0.0 | 18.0±0.0/0.0 | 16.7±1.2/7.0 |
| oxymetazoline hydrochloride | 3 | 16.0±1.0/11.0 | 15.7±0.6/13.0 | 13.0±3.5/28.0 |
| carvedilol | 3 | 18.0±0.0/0.0 | 17.0±1.0/6.0 | 16.7±1.2/7.0 |
| dexibuprofen | 3 | 17.0±1.0/6.0 | 16.3±0.6/9.0 | 14.3±2.1/20.0 |
| l-tryptophan | 3 | 18.0±0.0/0.0 | 15.3±0.6/15.0 | 14.7±0.6/19.0 |
| atropine sulfate monohydrate | 3 | 18.0±0.0/0.0 | 18.0±0.0/0.0 | 16.7±1.2/7.0 |
| linezolid | 3 | 18.0±0.0/0.0 | 16.7±1.2/7.0 | 15.3±0.6/15.0 |
| morantel tartrate | 3 | 18.0±0.0/0.0 | 18.0±0.0/0.0 | 18.0±0.0/0.0 |
| carbetapentane citrate | 3 | 18.0±0.0/0.0 | 18.0±0.0/0.0 | 16.7±1.5/7.0 |
| sulfinpyrazone | 3 | 18.0±0.0/0.0 | 17.3±0.6/4.0 | 17.0±1.0/6.0 |
| sodium gluconate | 3 | 18.0±0.0/0.0 | 18.0±0.0/0.0 | 18.0±0.0/0.0 |
| mecarbinate | 3 | 17.0±0.0/6.0 | 17.0±0.0/6.0 | 17.0±0.0/6.0 |
| pentoxifylline | 3 | 18.0±0.0/0.0 | 16.7±1.2/7.0 | 15.0±0.0/17.0 |
| valethamate bromide | 3 | 18.0±0.0/0.0 | 17.0±1.0/6.0 | 16.7±1.2/7.0 |
| toremifene | 3 | 17.0±1.0/6.0 | 17.0±1.0/6.0 | 16.3±0.6/9.0 |
| streptomycin sulfate | 3 | 18.0±0.0/0.0 | 15.3±0.6/15.0 | 14.7±0.6/19.0 |
| alfuzosin hydrochloride | 3 | 18.0±0.0/0.0 | 18.0±0.0/0.0 | 16.7±1.2/7.0 |
| zalcitabine | 3 | 18.0±0.0/0.0 | 16.7±1.2/7.0 | 15.3±0.6/15.0 |
| physostigmine salicylate | 3 | 18.0±0.0/0.0 | 18.0±0.0/0.0 | 16.7±1.2/7.0 |
| clemastine fumarate | 3 | 18.0±0.0/0.0 | 16.7±1.5/7.0 | 11.0±3.5/39.0 |
| pantoprazole sodium hydrate | 3 | 18.0±0.0/0.0 | 18.0±0.0/0.0 | 17.0±1.0/6.0 |
| homatropine bromide | 3 | 18.0±0.0/0.0 | 18.0±0.0/0.0 | 16.7±1.2/7.0 |
| pemetrexed acid | 3 | 16.0±1.0/11.0 | 15.7±0.6/13.0 | 15.3±0.6/15.0 |
| meptazinol hydrochloride | 3 | 18.0±0.0/0.0 | 18.0±0.0/0.0 | 18.0±0.0/0.0 |
| thalidomide | 3 | 17.3±0.6/4.0 | 15.0±1.0/17.0 | 13.7±0.6/24.0 |
| methyl hesperidin | 3 | 18.0±0.0/0.0 | 18.0±0.0/0.0 | 18.0±0.0/0.0 |
| mycophenolate mofetil | 3 | 18.0±0.0/0.0 | 17.0±1.0/6.0 | 16.7±1.2/7.0 |
| clomipramine hydrochloride | 3 | 18.0±0.0/0.0 | 16.3±0.6/9.0 | 14.3±0.6/20.0 |
| nilutamide | 3 | 18.0±0.0/0.0 | 15.3±0.6/15.0 | 14.7±0.6/19.0 |
| bephenium hydroxynaphthoate | 3 | 18.0±0.0/0.0 | 18.0±0.0/0.0 | 16.7±1.2/7.0 |
| triflupromazine hydrochloride | 3 | 18.0±0.0/0.0 | 16.7±1.2/7.0 | 15.3±0.6/15.0 |
| oxantel pamoate | 3 | 18.0±0.0/0.0 | 18.0±0.0/0.0 | 18.0±0.0/0.0 |
| fendiline hydrochloride | 3 | 18.0±0.0/0.0 | 17.7±0.6/2.0 | 16.7±1.5/7.0 |
| risperidal | 3 | 18.0±0.0/0.0 | 18.0±0.0/0.0 | 17.0±1.0/6.0 |
| sildenafil citrate | 3 | 18.0±0.0/0.0 | 18.0±0.0/0.0 | 16.7±1.2/7.0 |
| deflazacort | 3 | 18.0±0.0/0.0 | 15.7±0.6/13.0 | 15.3±0.6/15.0 |
| tyloxapol | 3 | 18.0±0.0/0.0 | 16.7±1.2/7.0 | 14.7±0.6/19.0 |
| vitamin e acetate | 3 | 17.0±0.0/6.0 | 16.0±0.0/11.1 | 15.7±0.6/13.0 |
| pempidine | 3 | 18.0±0.0/0.0 | 18.0±0.0/0.0 | 18.0±0.0/0.0 |
| aconitine | 3 | 18.0±0.0/0.0 | 18.0±0.0/0.0 | 18.0±0.0/0.0 |
| inosine | 3 | 18.0±0.0/0.0 | 17.0±1.0/6.0 | 16.7±1.2/7.0 |
| proflavine hemisulfate | 3 | 17.0±1.0/6.0 | 16.3±0.6/9.0 | 15.0±0.0/17.0 |
| glucosamine | 3 | 18.0±0.0/0.0 | 15.3±0.6/15.0 | 14.7±0.6/19.0 |
| folinic acid calcium salt pentahydrate | 3 | 18.0±0.0/0.0 | 18.0±0.0/0.0 | 16.7±1.2/7.0 |
| bicalutamide | 3 | 18.0±0.0/0.0 | 18.0±0.0/0.0 | 17.3±0.6/4.0 |
| loxoprofen | 3 | 18.0±0.0/0.0 | 18.0±0.0/0.0 | 16.3±1.5/9.0 |
| econazole | 3 | 18.0±0.0/0.0 | 18.0±0.0/0.0 | 18.0±0.0/0.0 |
| pefloxacin mesylate | 3 | 18.0±0.0/0.0 | 17.3±0.6/4.0 | 17.0±1.0/6.0 |
| edaravone | 3 | 18.0±0.0/0.0 | 18.0±0.0/0.0 | 16.7±1.2/7.0 |
| etamsylate | 3 | 16.0±1.0/11.0 | 15.7±0.6/13.0 | 15.3±1.0/22.0 |
| quinestrol | 3 | 18.0±0.0/0.0 | 16.7±1.2/7.0 | 15.0±0.0/17.0 |
| primidone | 3 | 18.0±0.0/0.0 | 16.0±0.0/11.1 | 15.7±0.6/13.0 |
| 4-aminoantipyrine | 3 | 18.0±0.0/0.0 | 18.0±0.0/0.0 | 18.0±0.0/0.0 |
| clofibric acid | 3 | 18.0±0.0/0.0 | 17.0±1.0/6.0 | 16.7±1.2/7.0 |
| antazoline hydrochloride | 3 | 18.0±0.0/0.0 | 16.3±0.6/9.0 | 16.3±0.6/9.0 |
| diltiazem hydrochloride | 3 | 18.0±0.0/0.0 | 18.0±0.0/0.0 | 18.0±0.0/0.0 |
| cefdinir | 3 | 18.0±0.0/0.0 | 18.0±0.0/0.0 | 18.0±0.0/0.0 |
| calcium folinate | 3 | 18.0±0.0/0.0 | 16.7±1.2/7.0 | 15.3±0.6/15.0 |
| quetiapin | 3 | 18.0±0.0/0.0 | 18.0±0.0/0.0 | 16.7±1.2/7.0 |
| saxagliptin hydrate | 3 | 18.0±0.0/0.0 | 16.7±1.5/7.0 | 14.7±1.5/19.0 |
| linagliptin | 3 | 18.0±0.0/0.0 | 17.3±0.6/4.0 | 17.0±1.0/6.0 |
| sulbentine | 3 | 18.0±0.0/0.0 | 18.0±0.0/0.0 | 18.0±0.0/0.0 |
| pioglitazone | 3 | 18.0±0.0/0.0 | 18.0±0.0/0.0 | 17.0±0.0/6.0 |
| rivastigmine | 3 | 18.0±0.0/0.0 | 17.0±1.0/6.0 | 16.7±1.2/7.0 |
| orciprenaline sulfate | 3 | 18.0±0.0/0.0 | 18.0±0.0/0.0 | 17.7±0.6/2.0 |
| lithium citrate tribasic tetrahydrate | 3 | 18.0±0.0/0.0 | 18.0±0.0/0.0 | 18.0±0.0/0.0 |
| cinoxacin | 3 | 18.0±0.0/0.0 | 18.0±0.0/0.0 | 18.0±0.0/0.0 |
| hexamethonium bromide | 3 | 18.0±0.0/0.0 | 16.7±1.2/7.0 | 15.3±0.6/15.0 |
| methylprodnisolone sodium succinate | 3 | 18.0±0.0/0.0 | 18.0±0.0/0.0 | 18.0±0.0/0.0 |
| isoxsuprine hydrochloride | 3 | 18.0±0.0/0.0 | 18.0±0.0/0.0 | 16.7±1.5/7.0 |
| prednisolone-21-acetate | 3 | 18.0±0.0/0.0 | 17.3±0.6/4.0 | 17.0±1.0/6.0 |
| osalmid | 3 | 18.0±0.0/0.0 | 18.0±0.0/0.0 | 18.0±0.0/0.0 |
| xylocaine | 3 | 18.0±0.0/0.0 | 15.7±0.6/13.0 | 15.3±0.6/15.0 |
| irinotecan hydrochloride trihydrate | 3 | 18.0±0.0/0.0 | 16.7±1.2/7.0 | 12.7±1.2/29.4 |
| etidronate | 3 | 18.0±0.0/0.0 | 18.0±0.0/0.0 | 18.0±0.0/0.0 |
| vitamin e | 3 | 17.0±1.0/6.0 | 16.3±0.6/9.0 | 16.3±0.6/9.0 |
| epirubicin hydrochloride | 3 | 18.0±0.0/0.0 | 16.0±1.0/11.0 | 14.7±0.6/19.0 |
| brucin | 3 | 18.0±0.0/0.0 | 18.0±0.0/0.0 | 16.7±1.2/7.0 |
| piracetam | 3 | 18.0±0.0/0.0 | 18.0±0.0/0.0 | 15.3±0.6/15.0 |
| alverine citrate | 3 | 18.0±0.0/0.0 | 18.0±0.0/0.0 | 16.7±1.2/7.0 |
| testosterone propionate | 3 | 18.0±0.0/0.0 | 18.0±0.0/0.0 | 18.0±0.0/0.0 |
| nitroxoline | 3 | 18.0±0.0/0.0 | 17.3±0.6/4.0 | 16.0±1.0/11.0 |
| erlotinib | 3 | 18.0±0.0/0.0 | 18.0±0.0/0.0 | 18.0±0.0/0.0 |
| phenylephrine hydrochloride | 3 | 16.0±1.0/11.0 | 15.7±0.6/13.0 | 13.3±1.5/26.0 |
| dicurone | 3 | 18.0±0.0/0.0 | 16.7±1.2/7.0 | 15.0±0.0/17.0 |
| guanidine hydrochloride | 3 | 17.0±0.0/6.0 | 16.0±0.0/11.1 | 16.0±0.0/11.1 |
| tetryzoline | 3 | 18.0±0.0/0.0 | 18.0±0.0/0.0 | 18.0±0.0/0.0 |
| sodium nitroprusside | 3 | 18.0±0.0/0.0 | 17.0±1.0/6.0 | 16.7±1.2/7.0 |
| dapoxetine hydrochloride | 3 | 18.0±0.0/0.0 | 16.3±0.6/9.0 | 15.7±0.6/13.0 |
| haloperidol | 3 | 18.0±0.0/0.0 | 15.3±0.6/15.0 | 14.7±0.6/19.0 |
| urethane | 3 | 18.0±0.0/0.0 | 18.0±0.0/0.0 | 18.0±0.0/0.0 |
| oxiniacic acid | 3 | 18.0±0.0/0.0 | 16.7±1.2/7.0 | 15.3±0.6/15.0 |
| budesonide | 3 | 18.0±0.0/0.0 | 18.0±0.0/0.0 | 18.0±0.0/0.0 |
| mebhydrolin napadisylate | 3 | 18.0±0.0/0.0 | 18.0±0.0/0.0 | 16.7±1.5/7.0 |
| estradiol valerate | 3 | 18.0±0.0/0.0 | 18.0±0.0/0.0 | 18.0±0.0/0.0 |
| bosutinib | 3 | 18.0±0.0/0.0 | 18.0±0.0/0.0 | 16.7±1.2/7.0 |
| argatroban | 3 | 18.0±0.0/0.0 | 18.0±0.0/0.0 | 18.0±0.0/0.0 |
| ticagrelor | 3 | 18.0±0.0/0.0 | 17.7±1.0/6.0 | 16.7±1.2/7.0 |
| levetiracetam | 3 | 17.0±1.0/6.0 | 16.3±0.6/9.0 | 14.3±0.6/20.0 |
| protriptyline hydrochloride | 3 | 18.0±0.0/0.0 | 15.3±0.6/15.0 | 14.7±0.6/19.0 |
| losartan potassium | 3 | 18.0±0.0/0.0 | 18.0±0.0/0.0 | 18.0±0.0/0.0 |
| prasugrel | 3 | 18.0±0.0/0.0 | 16.7±1.2/7.0 | 15.3±0.6/15.0 |
| acarbose | 3 | 18.0±0.0/0.0 | 18.0±0.0/0.0 | 18.0±0.0/0.0 |
| chromocarbe | 3 | 18.0±0.0/0.0 | 16.7±1.5/7.0 | 16.7±1.5/7.0 |
| meticrane | 3 | 18.0±0.0/0.0 | 18.0±0.0/0.0 | 17.0±1.0/6.0 |
| procodazole | 3 | 18.0±0.0/0.0 | 18.0±0.0/0.0 | 16.7±1.2/7.0 |
| digitoxin | 3 | 16.0±1.0/11.0 | 15.7±0.6/13.0 | 15.0±0.0/17.0 |
| buflomedil hydrochloride | 3 | 18.0±0.0/0.0 | 16.7±1.2/7.0 | 14.3±2.1/20.0 |
| carvedilol phosphate | 3 | 17.0±0.0/6.0 | 16.0±0.0/11.1 | 15.7±0.6/13.0 |
| canrenone | 3 | 18.0±0.0/0.0 | 18.0±0.0/0.0 | 18.0±0.0/0.0 |
| (+,-)-octopamine hydrochloride | 3 | 18.0±0.0/0.0 | 17.0±1.0/6.0 | 16.7±1.2/7.0 |
| metoprolol tartrate | 3 | 18.0±0.0/0.0 | 18.0±0.0/0.0 | 18.0±0.0/0.0 |
| mk3102 | 3 | 18.0±0.0/0.0 | 15.3±0.6/15.0 | 14.7±0.6/19.0 |
| corvotone | 3 | 18.0±0.0/0.0 | 18.0±0.0/0.0 | 16.7±1.2/7.0 |
| vitamin k1 | 3 | 18.0±0.0/0.0 | 16.7±1.2/7.0 | 15.3±0.6/15.0 |
| zopiclone | 3 | 18.0±0.0/0.0 | 18.0±0.0/0.0 | 18.0±0.0/0.0 |
| armodafinil | 3 | 18.0±0.0/0.0 | 18.0±0.0/0.0 | 16.7±1.5/7.0 |
| phthalylsulfacetamide | 3 | 18.0±0.0/0.0 | 17.3±0.6/4.0 | 17.0±1.0/6.0 |
| calcium gluconate | 3 | 18.0±0.0/0.0 | 18.0±0.0/0.0 | 16.7±1.2/7.0 |
| risedronic acid | 3 | 18.0±0.0/0.0 | 15.7±0.6/13.0 | 15.3±0.6/15.0 |
| berberine hydrochloride | 3 | 18.0±0.0/0.0 | 17.0±1.0/6.0 | 16.7±1.2/7.0 |
| salicoside | 3 | 17.0±1.0/6.0 | 16.3±0.6/9.0 | 16.0±0.0/11.0 |
| bismuth subsalicylat | 3 | 18.0±0.0/0.0 | 15.3±0.6/15.0 | 15.3±0.6/15.0 |
| acetanilide | 3 | 18.0±0.0/0.0 | 18.0±0.0/0.0 | 16.7±1.2/7.0 |
| estradiol benzoate | 3 | 18.0±0.0/0.0 | 16.7±1.2/7.0 | 15.3±0.6/15.0 |
| ferrous fumarate | 3 | 18.0±0.0/0.0 | 18.0±0.0/0.0 | 18.0±0.0/0.0 |
| doxapram hydrochloride | 3 | 18.0±0.0/0.0 | 16.7±1.5/7.0 | 15.7±0.6/13.0 |
| sulfalen | 3 | 18.0±0.0/0.0 | 17.3±0.6/4.0 | 17.0±1.0/6.0 |
| hydroxyzine pamoate | 3 | 18.0±0.0/0.0 | 18.0±0.0/0.0 | 18.0±0.0/0.0 |
| fluphenazine hydrochloride | 3 | 16.0±1.0/11.0 | 16.0±1.0/11.0 | 14.7±0.6/19.0 |
| ipratropium bromide monohydrate | 3 | 18.0±0.0/0.0 | 18.0±0.0/0.0 | 15.0±1.0/17.0 |
| balofloxacin | 3 | 18.0±0.0/0.0 | 18.0±0.0/0.0 | 18.0±0.0/0.0 |
| mirtazapine | 3 | 18.0±0.0/0.0 | 17.0±1.0/6.0 | 16.7±1.2/7.0 |
| oxaliplatin | 3 | 17.0±1.0/6.0 | 16.3±0.6/9.0 | 16.3±0.6/9.0 |
| nisoldipine | 3 | 18.0±0.0/0.0 | 18.0±0.0/0.0 | 18.0±0.0/0.0 |
| benazepril hydrochloride | 3 | 18.0±0.0/0.0 | 18.0±0.0/0.0 | 18.0±0.0/0.0 |
| fenoprofen calcium | 3 | 18.0±0.0/0.0 | 16.7±1.2/7.0 | 15.7±0.6/13.0 |
| brompheniramine hydrogen maleate | 3 | 18.0±0.0/0.0 | 18.0±0.0/0.0 | 18.0±0.0/0.0 |
| eletriptan hbr | 3 | 18.0±0.0/0.0 | 18.0±0.0/0.0 | 18.0±0.0/0.0 |
| proparacaine hydrochloride | 3 | 18.0±0.0/0.0 | 18.0±0.0/0.0 | 17.0±1.0/6.0 |
| clorprenaline hydrochloride | 3 | 18.0±0.0/0.0 | 18.0±0.0/0.0 | 16.7±1.2/7.0 |
| amcinonide | 3 | 18.0±0.0/0.0 | 18.0±0.0/0.0 | 18.0±0.0/0.0 |
| hydrocortisone valerate | 3 | 18.0±0.0/0.0 | 18.0±0.0/0.0 | 18.0±0.0/0.0 |
| aminacrine | 3 | 18.0±0.0/0.0 | 16.0±0.0/11.1 | 15.7±0.6/13.0 |
| methylthiouracil | 3 | 18.0±0.0/0.0 | 18.0±0.0/0.0 | 18.0±0.0/0.0 |
| diperodon hydrochloride | 3 | 18.0±0.0/0.0 | 17.0±1.0/6.0 | 16.7±1.2/7.0 |
| nimodipine | 3 | 17.0±1.0/6.0 | 16.3±0.6/9.0 | 15.3±0.6/15.0 |
| valpramide | 3 | 18.0±0.0/0.0 | 15.3±0.6/15.0 | 14.7±0.6/19.0 |
| nefopam hydrochloride | 3 | 18.0±0.0/0.0 | 17.3±0.6/4.0 | 16.7±1.2/7.0 |
| finasteride | 3 | 18.0±0.0/0.0 | 18.0±0.0/0.0 | 15.3±0.6/15.0 |
| trans-anethole | 3 | 18.0±0.0/0.0 | 18.0±0.0/0.0 | 18.0±0.0/0.0 |
| dithiopropanol | 3 | 18.0±0.0/0.0 | 18.0±0.0/0.0 | 18.0±0.0/0.0 |
| citicoline | 3 | 18.0±0.0/0.0 | 17.3±0.6/4.0 | 17.0±1.0/6.0 |
| strychnine | 3 | 18.0±0.0/0.0 | 18.0±0.0/0.0 | 16.7±1.2/7.0 |
| dehydrocholic acid | 3 | 16.0±1.0/11.0 | 15.7±0.6/13.0 | 15.7±0.6/13.0 |
| prochlorperazine maleate | 3 | 18.0±0.0/0.0 | 17.0±1.0/6.0 | 16.7±1.2/7.0 |
| dabigatran etexilate | 3 | 15.3±0.6/15.0 | 15.0±0.0/17.0 | 13.7±0.6/24.0 |
| pramoxine hydrochloride | 3 | 18.0±0.0/0.0 | 18.0±0.0/0.0 | 18.0±0.0/0.0 |
| dropropizine | 3 | 18.0±0.0/0.0 | 18.0±0.0/0.0 | 16.7±1.2/7.0 |
| artesunate | 3 | 18.0±0.0/0.0 | 16.7±1.2/7.0 | 15.3±0.6/15.0 |
| flopropione | 3 | 18.0±0.0/0.0 | 18.0±0.0/0.0 | 18.0±0.0/0.0 |
| pipemidic acid | 3 | 18.0±0.0/0.0 | 18.0±0.0/0.0 | 16.0±0.0/11.0 |
| eprosartan mesylate | 3 | 18.0±0.0/0.0 | 18.0±0.0/0.0 | 18.0±0.0/0.0 |
| acetylsalicylic acid | 3 | 18.0±0.0/0.0 | 18.0±0.0/0.0 | 18.0±0.0/0.0 |
| halazone | 3 | 16.0±1.0/11.0 | 15.7±0.6/13.0 | 15.3±0.6/15.0 |
| procaine | 3 | 18.0±0.0/0.0 | 16.7±1.2/7.0 | 15.0±2.7/17.0 |
| disulfiram | 3 | 18.0±0.0/0.0 | 17.0±1.0/6.0 | 16.7±1.2/7.0 |
| uracil | 3 | 17.0±1.0/6.0 | 16.3±0.6/9.0 | 16.3±0.6/9.0 |
| bendamustine hydrochloride | 3 | 18.0±0.0/0.0 | 18.0±0.0/0.0 | 18.0±0.0/0.0 |
| suplatast tosilate | 3 | 18.0±0.0/0.0 | 18.0±0.0/0.0 | 18.0±0.0/0.0 |
| cefpiramide acid | 3 | 18.0±0.0/0.0 | 16.7±1.2/7.0 | 15.7±0.6/13.0 |
| nebivolol hydrochloride | 3 | 18.0±0.0/0.0 | 18.0±0.0/0.0 | 18.0±0.0/0.0 |
| urapidil | 3 | 18.0±0.0/0.0 | 16.7±1.5/7.0 | 16.0±1.0/11.0 |
| loteprednol etabonate | 3 | 18.0±0.0/0.0 | 17.3±0.6/4.0 | 17.0±1.0/6.0 |
| chloroquine diphosphate | 3 | 18.0±0.0/0.0 | 18.0±0.0/0.0 | 18.0±0.0/0.0 |
| prednisolone phosphate sodium | 3 | 16.0±1.0/11.0 | 15.7±0.6/13.0 | 14.3±0.6/20.0 |
| bipariden | 3 | 18.0±0.0/0.0 | 18.0±0.0/0.0 | 18.0±0.0/0.0 |
| amlodipine besylate | 3 | 17.0±0.0/6.0 | 16.0±0.0/11.1 | 15.7±0.6/13.0 |
| acecainide | 3 | 18.0±0.0/0.0 | 18.0±0.0/0.0 | 18.0±0.0/0.0 |
| zomepirac sodium | 3 | 18.0±0.0/0.0 | 17.0±1.0/6.0 | 16.0±0.0/11.0 |
| escin | 3 | 18.0±0.0/0.0 | 18.0±0.0/0.0 | 18.0±0.0/0.0 |
| tizanidine hydrochloride | 3 | 18.0±0.0/0.0 | 15.3±0.6/15.0 | 14.7±0.6/19.0 |
| ipriflavone (osteofix) | 3 | 18.0±0.0/0.0 | 18.0±0.0/0.0 | 17.0±0.0/6.0 |
| dl-glutamine | 3 | 18.0±0.0/0.0 | 16.7±1.2/7.0 | 15.3±0.6/15.0 |
| estropipate | 3 | 18.0±0.0/0.0 | 18.0±0.0/0.0 | 18.0±0.0/0.0 |
| potassium iodide | 3 | 18.0±0.0/0.0 | 16.7±1.5/7.0 | 15.0±0.0/17.0 |
| venlafaxine hydrochloride | 3 | 18.0±0.0/0.0 | 18.0±0.0/0.0 | 17.0±1.0/6.0 |
| timolol maleate | 3 | 18.0±0.0/0.0 | 18.0±0.0/0.0 | 18.0±0.0/0.0 |
| ractopamine hydrochloride | 3 | 18.0±0.0/0.0 | 17.0±0.0/6.0 | 12.7±1.2/29.4 |
| crotamiton | 3 | 18.0±0.0/0.0 | 16.7±1.2/7.0 | 15.3±0.6/15.0 |
| phenethyl alcohol | 3 | 18.0±0.0/0.0 | 18.0±0.0/0.0 | 15.7±0.6/13.0 |
| dalbavancin hydrochloride | 3 | 18.0±0.0/0.0 | 18.0±0.0/0.0 | 18.0±0.0/0.0 |
| caffeine | 3 | 18.0±0.0/0.0 | 18.0±0.0/0.0 | 18.0±0.0/0.0 |
| anastrozole | 3 | 18.0±0.0/0.0 | 17.0±1.0/6.0 | 16.7±1.2/7.0 |
| cilnidipine | 3 | 18.0±0.0/0.0 | 18.0±0.0/0.0 | 16.3±0.6/9.0 |
| oxybuprocaine hydrochloride | 3 | 18.0±0.0/0.0 | 18.0±0.0/0.0 | 18.0±0.0/0.0 |
| minoxidil | 3 | 18.0±0.0/0.0 | 18.0±0.0/0.0 | 16.7±1.2/7.0 |
| diclofenac diethylamine | 3 | 18.0±0.0/0.0 | 16.7±1.2/7.0 | 15.3±0.6/15.0 |
| ethylenediaminetetraacetic acid trisodium salt solution | 3 | 18.0±0.0/0.0 | 18.0±0.0/0.0 | 18.0±0.0/0.0 |
| hexamethylenetetramine | 3 | 18.0±0.0/0.0 | 16.7±1.5/7.0 | 16.7±1.5/7.0 |
| atropine | 3 | 18.0±0.0/0.0 | 17.3±0.6/4.0 | 17.0±1.0/6.0 |
| mercaptopurine (6-mp) | 3 | 18.0±0.0/0.0 | 18.0±0.0/0.0 | 16.7±1.2/7.0 |
| salicylamide | 3 | 18.0±0.0/0.0 | 15.7±0.6/13.0 | 15.3±0.6/15.0 |
| enoxolone | 3 | 18.0±0.0/0.0 | 18.0±0.0/0.0 | 18.0±0.0/0.0 |
| bupivacaine hydrochloride | 3 | 18.0±0.0/0.0 | 16.0±0.0/11.1 | 15.7±0.6/13.0 |
| glafenine | 3 | 18.0±0.0/0.0 | 18.0±0.0/0.0 | 18.0±0.0/0.0 |
| folic acid | 3 | 18.0±0.0/0.0 | 17.0±1.0/6.0 | 16.7±1.2/7.0 |
| arbidol hydrochloride | 3 | 18.0±0.0/0.0 | 18.0±0.0/0.0 | 16.0±1.0/11.0 |
| nitrendipine | 3 | 18.0±0.0/0.0 | 18.0±0.0/0.0 | 17.0±1.0/6.0 |
| brinzolamide | 3 | 18.0±0.0/0.0 | 18.0±0.0/0.0 | 18.0±0.0/0.0 |
| rocuronium bromide | 3 | 18.0±0.0/0.0 | 16.7±1.2/7.0 | 15.0±0.0/17.0 |
| depofemin | 3 | 18.0±0.0/0.0 | 18.0±0.0/0.0 | 18.0±0.0/0.0 |
| clopidogrel | 3 | 18.0±0.0/0.0 | 16.7±1.5/7.0 | 16.7±1.5/7.0 |
| secnidazole | 3 | 18.0±0.0/0.0 | 18.0±0.0/0.0 | 17.0±0.0/6.0 |
| loperamide hydrochloride | 3 | 18.0±0.0/0.0 | 18.0±0.0/0.0 | 18.0±0.0/0.0 |
| valdecoxib | 3 | 18.0±0.0/0.0 | 18.0±0.0/0.0 | 18.0±0.0/0.0 |
| ethamivan | 3 | 18.0±0.0/0.0 | 18.0±0.0/0.0 | 18.0±0.0/0.0 |
| ciprofloxacin hydrochloride | 3 | 18.0±0.0/0.0 | 18.0±0.0/0.0 | 18.0±0.0/0.0 |
| merbromin | 3 | 18.0±0.0/0.0 | 18.0±0.0/0.0 | 18.0±0.0/0.0 |
| metaraminol bitartrate | 3 | 18.0±0.0/0.0 | 18.0±0.0/0.0 | 16.0±0.0/11.0 |
| meclocycline sulfosalicylate salt | 3 | 18.0±0.0/0.0 | 17.0±0.0/6.0 | 15.7±0.6/13.0 |
| evans blue | 3 | 18.0±0.0/0.0 | 18.0±0.0/0.0 | 16.7±1.2/7.0 |
| diphenhydramine hydrochloride | 3 | 18.0±0.0/0.0 | 18.0±0.0/0.0 | 18.0±0.0/0.0 |
| miconazole | 3 | 18.0±0.0/0.0 | 18.0±0.0/0.0 | 17.0±1.0/6.0 |
| aminoguanidine hydrochloride | 3 | 18.0±0.0/0.0 | 18.0±0.0/0.0 | 18.0±0.0/0.0 |
| arbutin | 3 | 18.0±0.0/0.0 | 16.0±0.0/11.1 | 15.3±0.6/15.0 |
| flutamide | 3 | 18.0±0.0/0.0 | 18.0±0.0/0.0 | 18.0±0.0/0.0 |
| hydroferulic acid | 3 | 18.0±0.0/0.0 | 17.0±1.0/6.0 | 16.7±1.2/7.0 |
| vigabatrin hydrochloride | 3 | 18.0±0.0/0.0 | 18.0±0.0/0.0 | 18.0±0.0/0.0 |
| 2-aminoethanethiol | 3 | 18.0±0.0/0.0 | 18.0±0.0/0.0 | 14.7±0.6/19.0 |
| sibutramine | 3 | 18.0±0.0/0.0 | 18.0±0.0/0.0 | 17.3±0.6/4.0 |
| aminophenazone | 3 | 18.0±0.0/0.0 | 18.0±0.0/0.0 | 18.0±0.0/0.0 |
| ropivacaine hydrochloride | 3 | 18.0±0.0/0.0 | 18.0±0.0/0.0 | 18.0±0.0/0.0 |
| clofazimine | 3 | 18.0±0.0/0.0 | 16.7±1.5/7.0 | 16.7±1.5/7.0 |
| gliquidone | 3 | 18.0±0.0/0.0 | 18.0±0.0/0.0 | 17.0±1.0/6.0 |
| pasiniazid | 3 | 18.0±0.0/0.0 | 18.0±0.0/0.0 | 18.0±0.0/0.0 |
| talc | 3 | 18.0±0.0/0.0 | 15.7±0.6/13.0 | 15.3±0.6/15.0 |
| d-mannitol | 3 | 18.0±0.0/0.0 | 16.7±1.2/7.0 | 15.0±0.0/17.0 |
| fenspiride hydrochloride | 3 | 17.0±0.0/6.0 | 16.0±0.0/11.1 | 14.7±0.6/19.0 |
| celecoxib | 3 | 18.0±0.0/0.0 | 18.0±0.0/0.0 | 18.0±0.0/0.0 |
| flubendazole | 3 | 18.0±0.0/0.0 | 17.0±1.0/6.0 | 16.7±1.2/7.0 |
| heptaminol hydrochloride | 3 | 17.0±1.0/6.0 | 16.3±0.6/9.0 | 16.3±0.6/9.0 |
| dienestrol | 3 | 18.0±0.0/0.0 | 15.3±0.6/15.0 | 14.7±0.6/19.0 |
| procainamide hydrochloride | 3 | 18.0±0.0/0.0 | 18.0±0.0/0.0 | 15.7±1.2/13.0 |
| oxeladin citrate | 3 | 18.0±0.0/0.0 | 16.7±1.2/7.0 | 15.3±0.6/15.0 |
| isoleucine | 3 | 18.0±0.0/0.0 | 18.0±0.0/0.0 | 16.7±1.2/7.0 |
| carbachol | 3 | 18.0±0.0/0.0 | 18.0±0.0/0.0 | 18.0±0.0/0.0 |
| azelnidipine | 3 | 18.0±0.0/0.0 | 18.0±0.0/0.0 | 18.0±0.0/0.0 |
| imiquimod | 3 | 18.0±0.0/0.0 | 18.0±0.0/0.0 | 18.0±0.0/0.0 |
| roxatidine acetate hydrochloride | 3 | 18.0±0.0/0.0 | 18.0±0.0/0.0 | 16.0±1.0/11.0 |
| norethynodrel | 3 | 18.0±0.0/0.0 | 16.7±1.2/7.0 | 15.7±0.6/13.0 |
| pramiracetam | 3 | 17.0±0.0/6.0 | 16.0±0.0/11.1 | 14.3±0.6/13.0 |
| diciofenac | 3 | 18.0±0.0/0.0 | 18.0±0.0/0.0 | 18.0±0.0/0.0 |
| tamsulosin hydrochloride | 3 | 18.0±0.0/0.0 | 18.0±0.0/0.0 | 18.0±0.0/0.0 |
| thioproline | 3 | 18.0±0.0/0.0 | 18.0±0.0/0.0 | 16.7±1.2/7.0 |
| modaline sulfate | 3 | 17.0±1.0/6.0 | 17.0±1.0/6.0 | 15.0±1.0/17.0 |
| sitagliptin | 3 | 18.0±0.0/0.0 | 15.3±0.6/15.0 | 14.7±0.6/19.0 |
| quinidine hydrochloride | 3 | 18.0±0.0/0.0 | 18.0±0.0/0.0 | 17.0±1.0/6.0 |
| hydrocortisone butyrate | 3 | 18.0±0.0/0.0 | 18.0±0.0/0.0 | 18.0±0.0/0.0 |
| flurbiprofen | 3 | 18.0±0.0/0.0 | 18.0±0.0/0.0 | 16.7±1.2/7.0 |
| citiolone | 3 | 18.0±0.0/0.0 | 18.0±0.0/0.0 | 16.7±1.5/7.0 |
| moxifloxacin hydrochloride | 3 | 18.0±0.0/0.0 | 17.7±0.6/2.0 | 17.0±1.0/6.0 |
| camylofin | 3 | 18.0±0.0/0.0 | 18.0±0.0/0.0 | 18.0±0.0/0.0 |
| ticlopidine hydrochloride | 3 | 16.0±1.0/11.0 | 15.7±0.6/13.0 | 15.3±0.6/15.0 |
| crystal violet | 3 | 18.0±0.0/0.0 | 16.7±1.2/7.0 | 12.3±0.6/31.0 |
| abbolactone | 3 | 17.0±0.0/6.0 | 16.0±0.0/11.1 | 15.7±0.6/13.0 |
| ziprasidone hydrochloride | 3 | 18.0±0.0/0.0 | 18.0±0.0/0.0 | 18.0±0.0/0.0 |
| ferulic acid | 3 | 18.0±0.0/0.0 | 18.0±0.0/0.0 | 16.7±1.2/7.0 |
| paeonol | 3 | 17.0±1.0/6.0 | 17.0±1.0/6.0 | 16.3±0.6/9.0 |
| naringin | 3 | 18.0±0.0/0.0 | 15.3±0.6/15.0 | 14.7±0.6/19.0 |
| sennoside a | 3 | 18.0±0.0/0.0 | 18.0±0.0/0.0 | 16.7±1.2/7.0 |
| dirithromycin | 3 | 18.0±0.0/0.0 | 16.7±1.2/7.0 | 12.0±0.0/33.0 |
| isoniazid | 3 | 18.0±0.0/0.0 | 18.0±0.0/0.0 | 18.0±0.0/0.0 |
| pyridoxine | 3 | 18.0±0.0/0.0 | 18.0±0.0/0.0 | 18.0±0.0/0.0 |
| floxuridine | 3 | 18.0±0.0/0.0 | 17.3±0.6/4.0 | 17.0±1.0/6.0 |
| scopolamine hbr trihydrate | 3 | 18.0±0.0/0.0 | 18.0±0.0/0.0 | 18.0±0.0/0.0 |
| ropivacaine | 3 | 18.0±0.0/0.0 | 18.0±0.0/0.0 | 15.7±0.6/13.0 |
| (r)-(+)-atenolol | 3 | 18.0±0.0/0.0 | 17.0±1.0/6.0 | 16.7±1.2/7.0 |
| oxybutynin | 3 | 18.0±0.0/0.0 | 17.3±0.6/4.0 | 16.3±1.2/9.0 |
| dehydroepiandrosterone | 3 | 18.0±0.0/0.0 | 18.0±0.0/0.0 | 18.0±0.0/0.0 |
| pioglitazone hydrochloride | 3 | 18.0±0.0/0.0 | 18.0±0.0/0.0 | 16.0±1.7/11.0 |
| fluoxetine hydrochloride | 3 | 18.0±0.0/0.0 | 16.7±1.2/7.0 | 15.3±0.6/15.0 |
| glafenine hydrochloride | 3 | 18.0±0.0/0.0 | 18.0±0.0/0.0 | 16.3±2.0/9.0 |
| tacrine hydrochloride hydrate | 3 | 18.0±0.0/0.0 | 16.7±1.5/7.0 | 16.7±1.5/7.0 |
| vardenafil hydrochloride trihydrate | 3 | 18.0±0.0/0.0 | 17.3±0.6/4.0 | 17.0±1.0/6.0 |
| dasatinib monohydrate | 3 | 18.0±0.0/0.0 | 18.0±0.0/0.0 | 16.7±1.2/7.0 |
| cyproheptadine hydrochloride | 3 | 16.0±1.0/11.0 | 15.7±0.6/13.0 | 15.3±0.6/15.0 |
| amiloride hydrochloride dihydrate | 3 | 18.0±0.0/0.0 | 18.0±0.0/0.0 | 18.0±0.0/0.0 |
| actarit | 3 | 18.0±0.0/0.0 | 17.0±1.0/6.0 | 16.7±1.2/7.0 |
| dextromethorphan hbr monohydrate | 3 | 18.0±0.0/0.0 | 18.0±0.0/0.0 | 18.0±0.0/0.0 |
| lapatinib ditosylate | 3 | 18.0±0.0/0.0 | 15.3±0.6/15.0 | 14.7±0.6/19.0 |
| cloperastine hydrochloride | 3 | 18.0±0.0/0.0 | 18.0±0.0/0.0 | 18.0±0.0/0.0 |
| ranolazine 2hydrochloride | 3 | 18.0±0.0/0.0 | 18.0±0.0/0.0 | 18.0±0.0/0.0 |
| dl-adrenaline hydrochloride | 3 | 18.0±0.0/0.0 | 18.0±0.0/0.0 | 18.0±0.0/0.0 |
| 2-aminobenzenesulfonamide | 3 | 18.0±0.0/0.0 | 18.0±0.0/0.0 | 16.7±1.5/7.0 |
| scopolamine n-oxide hbr | 3 | 18.0±0.0/0.0 | 17.3±0.6/4.0 | 17.0±1.0/6.0 |
| sunitinib | 3 | 18.0±0.0/0.0 | 18.0±0.0/0.0 | 18.0±0.0/0.0 |
| warfarin sodium | 3 | 18.0±0.0/0.0 | 18.0±0.0/0.0 | 15.3±0.6/15.0 |
| manidipine 2hydrochloride | 3 | 18.0±0.0/0.0 | 16.7±1.2/7.0 | 13.3±1.5/26.0 |
| sibutramine hydrochloride monohydrate | 3 | 17.0±0.0/6.0 | 16.0±0.0/11.1 | 15.7±0.6/13.0 |
| lidocaine hydrochloride hydrate | 3 | 18.0±0.0/0.0 | 18.0±0.0/0.0 | 18.0±0.0/0.0 |
| bengenin | 3 | 18.0±0.0/0.0 | 17.0±1.0/6.0 | 16.7±1.2/7.0 |
| 17-beta-estradiol-3,17-dipropionate | 3 | 17.0±1.0/6.0 | 16.3±0.6/9.0 | 16.3±0.6/9.0 |
| losartan | 3 | 18.0±0.0/0.0 | 15.3±0.6/15.0 | 14.7±0.6/19.0 |
| metyrapone | 3 | 18.0±0.0/0.0 | 18.0±0.0/0.0 | 18.0±0.0/0.0 |
| camphor | 3 | 18.0±0.0/0.0 | 18.0±0.0/0.0 | 15.3±0.6/15.0 |
| protocatechuic acid | 3 | 18.0±0.0/0.0 | 18.0±0.0/0.0 | 16.7±1.2/7.0 |
| glycyrrhizin (glycyrrhizic acid) | 3 | 18.0±0.0/0.0 | 16.7±1.5/7.0 | 16.7±1.5/7.0 |
| l-menthol | 3 | 18.0±0.0/0.0 | 18.0±0.0/0.0 | 17.0±1.0/6.0 |
| xylitol | 3 | 18.0±0.0/0.0 | 18.0±0.0/0.0 | 16.7±1.2/7.0 |
| 4-hydroxyantipyrine | 3 | 18.0±0.0/0.0 | 18.0±0.0/0.0 | 18.0±0.0/0.0 |
| sulfanilamide | 3 | 18.0±0.0/0.0 | 18.0±0.0/0.0 | 18.0±0.0/0.0 |
| theophylline monohydrate | 3 | 17.0±0.0/6.0 | 16.0±0.0/11.1 | 15.7±0.6/13.0 |
| chloropyramine hydrochloride | 3 | 18.0±0.0/0.0 | 18.0±0.0/0.0 | 18.0±0.0/0.0 |
| iodoquinol | 3 | 18.0±0.0/0.0 | 18.0±0.0/0.0 | 18.0±0.0/0.0 |
| phenylindione | 3 | 18.0±0.0/0.0 | 17.0±1.0/6.0 | 16.7±1.2/7.0 |
| tioxolone | 3 | 17.0±1.0/6.0 | 14.3±1.5/20.0 | 13.0±2.7/28.0 |
| pancreatin | 3 | 18.0±0.0/0.0 | 18.0±0.0/0.0 | 18.0±0.0/0.0 |
| methacycline hydrochloride | 3 | 18.0±0.0/0.0 | 18.0±0.0/0.0 | 17.3±0.6/4.0 |
| euquinine | 3 | 18.0±0.0/0.0 | 18.0±0.0/0.0 | 18.0±0.0/0.0 |
| lenvatinib (e7080) | 3 | 18.0±0.0/0.0 | 18.0±0.0/0.0 | 18.0±0.0/0.0 |
| thimerosal | 3 | 18.0±0.0/0.0 | 16.7±1.5/7.0 | 15.0±1.0/17.0 |
| diammonium glycyrrhizinate | 3 | 18.0±0.0/0.0 | 17.3±0.6/4.0 | 17.0±1.0/6.0 |
| sodium tauroglycocholate | 3 | 18.0±0.0/0.0 | 18.0±0.0/0.0 | 18.0±0.0/0.0 |
| zileuton | 3 | 18.0±0.0/0.0 | 18.0±0.0/0.0 | 18.0±0.0/0.0 |
| progesterone | 3 | 18.0±0.0/0.0 | 16.7±1.2/7.0 | 16.7±1.2/7.0 |
| doxofylline | 3 | 17.0±0.0/6.0 | 16.0±0.0/11.1 | 14.7±1.2/19.0 |
| azilsartan kamedoxomil | 3 | 18.0±0.0/0.0 | 18.0±0.0/0.0 | 18.0±0.0/0.0 |
| chlorpropamide | 3 | 18.0±0.0/0.0 | 17.0±1.0/6.0 | 13.3±2.5/26.0 |
| dimethyl fumarate | 3 | 18.0±0.0/0.0 | 18.0±0.0/0.0 | 16.3±0.6/9.0 |
| tyrosine | 3 | 18.0±0.0/0.0 | 15.3±0.6/15.0 | 14.7±0.6/19.0 |
| tetramisole hydrochloride | 3 | 18.0±0.0/0.0 | 18.0±0.0/0.0 | 17.3±0.6/4.0 |
| aminothiazole | 3 | 18.0±0.0/0.0 | 16.7±1.2/7.0 | 14.3±1.5/20.0 |
| lauroseptol | 3 | 18.0±0.0/0.0 | 18.0±0.0/0.0 | 18.0±0.0/0.0 |
| sasapyrine | 3 | 18.0±0.0/0.0 | 16.7±1.5/7.0 | 16.7±1.5/7.0 |
| capitrol | 3 | 18.0±0.0/0.0 | 17.3±0.6/4.0 | 17.0±1.0/6.0 |
| leucobasal | 3 | 18.0±0.0/0.0 | 18.0±0.0/0.0 | 18.0±0.0/0.0 |
| zinc pyrithione | 3 | 18.0±0.0/0.0 | 16.0±1.0/11.0 | 15.3±0.6/15.0 |
| cyclandelate | 3 | 18.0±0.0/0.0 | 17.0±1.0/6.0 | 16.7±1.2/7.0 |
| l-(-)-α-methyldopa | 3 | 18.0±0.0/0.0 | 18.0±0.0/0.0 | 18.0±0.0/0.0 |
| clotrimazole | 3 | 18.0±0.0/0.0 | 18.0±0.0/0.0 | 18.0±0.0/0.0 |
| sulfaguanidine | 3 | 18.0±0.0/0.0 | 18.0±0.0/0.0 | 18.0±0.0/0.0 |
| tolperisone hydrochloride | 3 | 18.0±0.0/0.0 | 16.7±1.2/7.0 | 15.3±0.6/15.0 |
| phthalylsulfathiazole | 3 | 18.0±0.0/0.0 | 18.0±0.0/0.0 | 18.0±0.0/0.0 |
